# Developmental Dysfunction of the Central Nervous System Lymphatics Modulates the Adaptive Neuro-Immune Response in the Perilesional Cortex in a Mouse Model of Traumatic Brain Injury

**DOI:** 10.3389/fimmu.2020.559810

**Published:** 2021-01-27

**Authors:** Sara Wojciechowski, Anaïs Virenque, Maria Vihma, Barbara Galbardi, Erin Jane Rooney, Meike Hedwig Keuters, Salli Antila, Jari Koistinaho, Francesco M. Noe

**Affiliations:** ^1^ A.I. Virtanen Institute for Molecular Sciences, University of Eastern Finland, Kuopio, Finland; ^2^ Neuroscience Center, Helsinki Institute of Life Science (HiLIFE), University of Helsinki, Helsinki, Finland; ^3^ Breast Cancer Unit, Department of Medical Oncology, IRCCS Ospedale San Raffaele, Milano, Italy; ^4^ Wihuri Research Institute and Translational Cancer Medicine Program, Faculty of Medicine, University of Helsinki, Helsinki, Finland

**Keywords:** controlled cortical impact (CCI), meningeal lymphatic vessels, CD8+ T lymphocytes, resident memory T cells, Chronic Traumatic Brain injury

## Abstract

**Rationale:**

The recently discovered meningeal lymphatic vessels (mLVs) have been proposed to be the missing link between the immune and the central nervous system. The role of mLVs in modulating the neuro-immune response following a traumatic brain injury (TBI), however, has not been analyzed. Parenchymal T lymphocyte infiltration has been previously reported as part of secondary events after TBI, suggestive of an adaptive neuro-immune response. The phenotype of these cells has remained mostly uncharacterized. In this study, we identified subpopulations of T cells infiltrating the perilesional areas 30 days post-injury (an early-chronic time point). Furthermore, we analyzed how the lack of mLVs affects the magnitude and the type of T cell response in the brain after TBI.

**Methods:**

TBI was induced in K14-VEGFR3-Ig transgenic (TG) mice or in their littermate controls (WT; wild type), applying a controlled cortical impact (CCI). One month after TBI, T cells were isolated from cortical areas ipsilateral or contralateral to the trauma and from the spleen, then characterized by flow cytometry. Lesion size in each animal was evaluated by MRI.

**Results:**

In both WT and TG-CCI mice, we found a prominent T cell infiltration in the brain confined to the perilesional cortex and hippocampus. The majority of infiltrating T cells were cytotoxic CD8+ expressing a CD44^hi^CD69+ phenotype, suggesting that these are effector resident memory T cells. K14-VEGFR3-Ig mice showed a significant reduction of infiltrating CD4+ T lymphocytes, suggesting that mLVs could be involved in establishing a proper neuro-immune response. Extension of the lesion (measured as lesion volume from MRI) did not differ between the genotypes. Finally, TBI did not relate to alterations in peripheral circulating T cells, as assessed one month after injury.

**Conclusions:**

Our results are consistent with the hypothesis that mLVs are involved in the neuro-immune response after TBI. We also defined the resident memory CD8+ T cells as one of the main population activated within the brain after a traumatic injury.

## Introduction

Traumatic brain injury (TBI) is among the top causes of death and disability in adult life (1, 2). It is estimated that at least 70 million people worldwide incur TBI every year (3). The number of prevalent cases (as reported for 2016) is above 55 million, with patients suffering from a wide range of lifelong physical and psychological invalidities (4).

TBI is defined as an alteration in brain function, or other evidence of brain pathology, caused by an external force (5), which results in immediate neuronal cell death, diffuse axonal injury, ischemia, and hemorrhage (6). These primary insults initiate a progressive cascade of secondary injuries, which include macrophage infiltration (7), neuro-inflammation (microglia and astrocyte activation associated with cytokine production), edema formation, oxidative stress, neuronal necrosis and apoptosis, and white matter atrophy (6). Secondary injuries can progress for years in patients and rodent models of TBI, and cause neurological and psychiatric deficits associated with the pathology (8).

Recruitment of peripheral immune cells, including T lymphocytes, into the brain is among the secondary events that have been described following TBI (9–12). Two distinct waves of infiltrating CD3+ T cells have been reported in the injured brain. First, a massive infiltration immediately commences after trauma and peaks 3 days post-injury (dpi) (9). After one month, there is a late adaptive immune response with a second recruitment, which persists chronically (11). However, the mechanisms and the consequences of the activation of the adaptive immune system after TBI are still poorly understood.

A proper immune surveillance of the brain was long disputed (13), due to the lack of a classical lymphatic system within the central nervous system (CNS). However, recent studies have described the presence of anatomically distinct lymphatic vessels in the meninges surrounding the brain and the spinal cord. These meningeal lymphatic vessels (mLVs) preferentially drain the cerebrospinal fluid together with cells and macromolecules into the deep cervical lymph nodes (dcLNs) (14–17). Within these secondary lymphoid organs, brain-derived antigens are presented to resident T lymphocytes, evoking different cellular fate and immune responses based on the inflammatory milieu. It has been demonstrated that dcLNs, together with superficial cervical LNs (scLNs), play a specific role in neuro-immune interaction, ensuring the protection of brain cells by promoting a non-cytotoxic immune response (18–20). From this prospective, mLVs and dcLNs are essential components of a putative specific CNS lymphatic system, and the mLVs could be essential in the activation of immune responses to brain insults.

The aim of our work is to better characterize the late adaptive immune response and to decipher the mechanisms underpinning the activation of T lymphocytes after TBI, focusing on the specific role of mLVs in this process. In this regard, we induced a cerebral contusion in the cortex of transgenic K14-VEGFR3-Ig (TG) mice that completely lack lymphatic vessels in several tissues, including the meninges (16, 21, 22). One month after brain injury, infiltrating T lymphocytes and circulating peripheral T cell populations in the spleen were phenotyped by flow cytometry. MRI was used to evaluate lesion size by comparing TG animals to their wild type (WT) littermates. We determined the persistence of putative resident memory cells mediating a CD8+ cytotoxic immune response in the perilesional cortical areas after TBI. We further demonstrate that a functional mLVs are important for the neuro-immune interaction after TBI, and the lack of mLVs results in the imbalance of the evoked T cell immune response. Our data also show that the TBI-elicited response in the CNS is specific, and that the analysis of the systemic immunity does not reflect the immune activation observed within the brain. No differences in MRI cortical lesion were found between the two genotypes. We suggest that the brain resident memory T cells, presenting an effector phenotype, are part of the cellular components characterizing the secondary injuries after TBI.

## Material and Methods

### Mice

Initial breeding pairs of K14-VEGFR3-Ig mice [C57BL/6JOlaHsd background (21)] were transferred from the University of Helsinki, and the colony was further expanded and maintained at University of Eastern Finland (Kuopio, Finland). Wild type and transgenic K14-VEGFR3-Ig mice used in all the experiments were littermates. Genotype screening was routinely confirmed by polymerase chain reaction analysis of ear punch samples. Mixed WT and TG mice were housed in standard laboratory cages (four animals per cage, until surgery) in a controlled enriched environment (constant temperature, 22 ± 1°C, humidity 50–60%, lights on 07:00–19:00), with food and water available *ad libitum* (23). After TBI induction, mice were kept two per cage, separated individually by a pierced partition. All animal procedures were approved by the Animal Ethics Committee of the Provincial Government of Southern Finland (ESAVI-2018-008787) and performed in accordance with the guidelines of the European Community Council Directives 2010/63/EU.

### Controlled Cortical Injury Mouse Model of Traumatic Brain Injury

All surgical procedures were performed aseptically whenever possible. Adult, 5 month-old male mice were deeply anesthetized with isoflurane (5% for induction, 1.0–1.5% for maintenance, in 0.5 L/min air; see **Supplementary Table 1**), injected with Carprofen (4 mg/Kg; s.c.) and the heads fixed to a stereotaxic frame (Kopf, Tujunga, USA). The scalp was shaved and then scrubbed (3x) with Betadine (povidone-iodine 10%) and 70% ethanol alternately, then local anesthesia of 2% Xylocain gel was applied. After skull exposure, a 5 mm circular craniotomy was manually drilled over the left parieto-temporal cortex, with the posterior edge of the craniotomy apposed to the lambdoid suture and the right edge to the sagittal suture. In order to reduce heating during manual craniotomy, the skull was irrigated with cold 0.9% saline solution. The carved bone was carefully removed, without disrupting the underlying dura, and placed in 1% Betadine solution. Thereafter, the animal was disconnected from isoflurane anesthesia for 5 min [stage 3 plane 1 according to Guedel’s classification (24)], and CCI was induced using an electromagnetic stereotaxic impact actuator (ImpactOne, Leica, Richmond, VA, USA). The 3 mm blunt tip of the impactor was adjusted to the center of the exposed dura perpendicular to the brain surface, and the impact was administered at a depth of 0.5 mm, speed of 5.0 m/s, and dwell time of 100 ms. The total duration of the craniotomy procedure including anesthesia induction was 35–40 min (**Supplementary Table 1**). After the impact, the mouse was reconnected to the isoflurane system and the skull secured with bone cement (Selectaplus + Palacos R+G 50/50). The scalp was sutured and treated with Cicatrene powder (Neomycin + Bacitracin) and Terramycin spray (Oxytetracycline). The total duration of post-impact surgery was 10 min. The mice were injected i.p. with 1 ml pre-warmed sterile saline (35°C) and allowed to fully recover in an incubator at 32°C. Mice were followed for the subsequent 48 h for any signs of illness or distress, in which case Carprofen was administered. Daily examination was performed for general health/mortality and morbidity for the rest of the study. No mortality was observed.

Craniotomy-related neuroinflammation has been previously reported in this model and the craniotomy itself (surgery) can be considered a form of mild brain trauma (25, 26). Moreover, CCI is a model of penetrating injury, involving dura damage, which has a severity that bypasses the possible effect of meningeal inflammation related to the craniotomy. The aim of our study is to characterize the adaptive immunity in response to a moderate TBI. Therefore, we did not analyze how differences in trauma severity (*i.e.*, CCI *vs.* sham-surgery) can affect the neuro-immune response. In compliance to the 3R principle, we excluded the sham-operated animals and used naïve mice not exposed to the surgical procedure as proper controls.

### *In Vivo* Magnetic Resonance Imaging and Lesion Volume Definition

MRI data were acquired 21 days after TBI induction in a 7T horizontal magnet (Bruker Pharmascan, Ettlingen, Germany). Images were acquired using a four-channel mouse brain surface coil, a 3D T2-weighted Fast Spin-Echo sequence (RARE, repetition time 1.5 s, effective echo time 48 ms, 16 echoes per excitation) with 100 µm isotropic resolution (field of view 25.6 mm x 128.8 mm x 9.6 mm; acquisition matrix 128 x 256 x 96). Scans were performed with the mouse under 1.0–1.5% maintenance isoflurane anesthesia (70/30 N_2_O/oxygen gas mixture, 1 L/min). The average acquisition time was 40 min, including anesthesia induction. A pressure sensor was used to monitor the respiratory rate, and respiratory gating was used to minimize motion artifacts.

T2-weighted images were used to evaluate the extent of the lesion (**Figure 5**, and **Supplementary Figures 4** and **5**). Regions of interest (ROIs) were outlined for volumetric analysis, avoiding the brain-skull interface and ventricles, throughout the entire extension of the brain (excluding olfactory bulbs and cerebellum). Lesion was defined as cortical/subcortical areas with hyper-intense signal (cystic lesion) and/or signal void areas (tissue cavity) from T2-weighted images (27, 28). Volumes of the lesion and of the ipsilateral and contralateral hemispheres were measured using Aedes (http://aedes.uef.fi), an in-house written MatLab program (MathWorks, Natick, MA). The lesion volume and the volumes of ipsilateral and contralateral healthy hemispheres were calculated from 80 consecutive slices in the coronal plane and adjusted in the sagittal plane (66 slices) and in the axial plane (99 slices) with a volume resolution of 200 x 500 x 100 µm.

### Quantification of Brain Contusion Area and Brain Atrophy

Measured volumes from MRI analysis were used to quantify the volume of the brain contusion and the brain atrophy, as previously described (29, 30). The relative percentage of infarct volume was calculated using the following formula:

$$contusion\;volume\;\left(\%\right)=\;\frac{Vc\;–\;\left(Vi\;–\;Vl\right)}{Vc}*100$$

and brain atrophy was determined as:

$$brain\;atrophy\;\left(\%\right)=\;\frac{\left(Vi\;–\;Vc\right)}{Vc}*100$$

where *Vc* = volume of contralateral hemisphere; *Vi* = volume of ipsilateral hemisphere; and *Vl* = measured lesion volume.

Analysis was performed blinded to the study groups. The contusion volume was measured from 22 TBI mice from the following experimental groups: WT–CCI, n = 13; and TG–CCI, n = 9. Analyses of contusion volume and brain atrophy progression at day 3 and day 14 has been conducted on *ad hoc* prepared mice: WT–CCI, n = 4; and TG–CCI, n = 4.

### Cell Isolation of Leukocytes

Thirty days after TBI induction, mice were anesthetized with an overdose of Avertin (Sigma, St. Louis, MO, USA) then transcardially perfused with ice-cold heparinized saline (6 min, 6 ml/min). Brains were collected and placed on ice in calcium and magnesium-free Hanks Balanced salt solution (HBSS) with 25 mM HEPES (both from Sigma).

Based on the analysis of MRI, we defined *a priori* the mean extension of the lesion and of the perilesional areas for all the TBI mice. Brains were sliced using a 1 mm scored matrix (Zivic Instruments, Pittsburgh, PA, USA): 6 mm thick coronal cut encompassing the lesion area was split along the central sagittal axis into left injured and right uninjured sides. Cortical areas enclosed between the rhinal and the sagittal sulci, and the corresponding hippocampi, were further isolated, pooled together, and placed in HBSS+HEPES. From the injured sides, penetrated cortical areas were visually identified (lesion area - **Supplementary Figure 1**) and carefully excised along the lesion ridge to pick only the perilesional cortex for further purification of leukocytes.

Brain samples were minced with scissors and then incubated at 37°C on a roller for 30 min in digest buffer containing 1.25 mg/ml Collagenase Type 4 (Worthington, Lakewood, NJ, USA) and 100 U/ml DNAseI (Sigma) in DMEM with GlutaMAX (Gibco Thermo Fisher Scientific, Waltham, MA, USA). Samples were filtered through a 100 µm cell strainer (Corning, Weisbaden, Germany), and centrifuged at 600 x g for 5 min. Myelin debris was removed using Debris Removal Solution (Miltenyi Biotech, Bergisch Gladbach, Germany) according to the manufacturer’s protocol. Briefly, cells were resuspended in ice-cold Dulbecco’s phosphate buffered saline (D-PBS, Sigma) with Debris Removal Solution, then overlaid with ice-cold D-PBS and centrifuged at 2,500 x g for 10 min at 4°C. Supernatant including myelin layer was carefully removed leaving the clear phase and the pellet. Samples were washed in ice-cold D-PBS, centrifuged at 600 x g for 10 min at 4°C, and the recovered pellets were stained directly for flow cytometry.

Spleens and dcLNs were separately collected in ice-cold HBSS+HEPES and each processed by crushing through a 70 µm cell strainer (Corning). dcLNS were washed with ice-cold D-PBS containing 1% bovine serum albumin (BSA) and 2 mM ethylenediaminetetraacetic acid (EDTA), centrifuged 500 x g for 10 min and resuspended in RPMI-1640 (all from Sigma). Crushed spleens were washed with ice-cold HBSS+HEPES, centrifuged 500 x g for 5 min before red blood cells were lysed in 1X PharmLyse (BD Biosciences, San Jose, CA USA) for 8 min at room temperature (RT). Lysed cells were washed with HBSS+HEPES, centrifuged as above, resuspended in RPMI-1640 (Sigma), and counted on a Bürker grid hemocytometer.

### Flow Cytometry Staining and Analysis

Spleen cells (500,000 per mouse), and total cells isolated from dcLNs and brain were each stained separately. Cells were first washed with D-PBS, and centrifuged at 400 x g for 5 min. The supernatant was removed, and then Zombie NIR fixable viability dye (1:1,000 BioLegend, San Diego, CA, USA) was added for 15 min at RT. Without washing, CD16/32 FcR block (5 µg/ml, BD Biosciences) was added followed by the appropriate antibody mix. Antibodies used: TCRβ PE-Cy7 (1:100 or 1:200 clone H57-597), CD44 PE (1:300 clone IM7) (both BioLegend); CD8a APC-R700 (1:150 or 1:200, clone 53-6.7), CD69 BV421 (1:100, clone H1.2F3), CD25 BB515 (1:150, clone PC61) (BD Biosciences); CD4 FITC (1:500, clone RM4-5), CD4 eFluor506 (1:500, clone RM4-5), CD8 PerCP eFluor710 (1:300, clone 53-6.7), CD44 APC (1:300 or 1:400, clone IM7), FoxP3 (1:40, clone FJK-16s) (eBioscience Thermo Fisher Scientific, Waltham, MA, USA); CD69 APC (1:20, clone H1.2F3, Miltenyi Biotech). All antibodies were used at titers determined empirically under experimental conditions.

Cells were incubated for 30 min at 4°C. Afterwards, samples were washed twice in HBSS with 1% FBS and then run on FACSAriaIII (BD Biosciences) equipped with 488 and 633 nm lasers, or on CytoFLEX S (Beckmann Coulter) equipped with 405, 488, 561, and 638 nm lasers, both with standard configuration. Compensations were made using OneComp and UltraComp Beads for antibody fluorescence (eBioscience Thermo Fisher Scientific) and ArC amine reactive beads for viability dye (Molecular Probes, Eugene, Oregon, USA). Fluorescent-Minus-One (FMO) controls were made to ensure gating. These control samples contained all antibodies except one to display fluorescent spreading error of compensated data in each channel (31). Data were analyzed using FCSExpress v5 (Denovo Software, Los Angeles, CA, USA) and FlowJo v10.4 (Treestar, Portland, OR, USA). The gating strategy used for the flow cytometry analysis of brain-isolated immune cells is reported in **Supplementary Figure 1**.

### CD3 Immunohistochemical Staining

Three mice per genotype were injured and sacrificed 30 days after TBI for the immunohistochemical (IHC) estimation of T lymphocyte localization in the brain. Mice were transcardially perfused with ice-cold NaCl 0.9% followed by 4% PFA. Brains were dissected and post-fixed in 4% PFA by immersion for 24 h at 4°C. Thereafter, specimens were cryoprotected by incubation in 20% glycerol [in 0.02 M potassium phosphate-buffered saline (KPBS), pH 7.4] for 48 h, frozen in N-pentane (3 min at -60°C), and stored at -70°C until sectioning. Frozen coronal sections were cut 25 µm with a sliding microtome, and collected in solution containing 30% ethylene glycol, 25% glycerol in 0.05 M phosphate buffer (PB) and stored at -20°C until further processing. Three sections per brain (approx. 700 µm apart, encompassing the antero-posterior extension of the lesion), were used to estimate the localization of CD3+ infiltrating T lymphocytes by IHC. Floating sections were washed in three changes of 1X PBS before being incubated for 1 h at RT in blocking solution [2% normal goat serum, 1% bovine serum albumin (BSA) 0.1% Triton X-100 and 0.05% Tween20 in PBS]. Sections were incubated overnight at 4°C with rat anti-mouse CD3ϵ (1:500, clone 17A2, eBioscience Thermo Fisher Scientific) and mouse anti-GFAP (1:500, Sigma) in staining buffer PBS with 1% BSA and 0.05% Triton X-100. After washing 3x with PBS, sections were incubated with conjugated goat secondary antibody anti-rat Alexa Fluor 647 and anti-mouse Alexa Fluor 546- in above staining buffer for 1 h at RT (Respectively 1:500 and 1:250, both Thermo Fisher Scientific). Finally, the sections were washed 3x in PBS and 10 min in 1X PB and mounted on Superfrost Plus slides (Thermo Scientific) with Vectashield containing DAPI (Vector Laboratories, Burlingame, CA, USA). Panoramic photomicrographs of the stained sections were captured using 20X objective with a fluorescence microscope (Zeiss Observer.Z1), and high-resolution Z-stack images were captured using 20X objective with a confocal microscope (Zeiss LSM710). ZEN 2012 software (Carl Zeiss GmbH) was used for image processing.

### Microtubule-Associated Protein 2, NeuN, and Glial Fibrillary Acidic Protein Staining and Analysis

Three sections located at bregma level +0,02, -2,06, and -4,04 mm (corresponding to the anterior and posterior edges and to the center of the lesion site) were selected from the previously sliced brains and stained for the Microtubule-Associated Protein 2 (MAP2; neuronal dendrites), the neuronal antigen NeuN, and the Glial Fibrillary Acidic Protein (GFAP; Type III intermediate filaments in astrocyte). For immunofluorescence procedure, sections were washed in blocking solution (4% BSA, 0,2% Triton X-100 in PBS) for 1 h at RT, followed by overnight incubation at 4°C with the following primary antibodies diluted in blocking solution: mouse anti-GFAP (1:500, Sigma G3893), guinea pig anti-NeuN (1:500, Millipore ABN90), rabbit anti-MAP2 (1:300, Abcam ab32454). After washing in PBS, sections were incubated for 2 h at RT with secondary fluorescent antibodies in blocking solution: Alexa Fluor 546-conjugated goat anti mouse (1:250), Alexa Fluor 488-conjugated goat anti rabbit (1:250), Alexa Fluor 633-conjugated goat anti guinea pig (1:200 all from Invitrogen, Thermo Fisher Scientific). Next, sections were washed in PBS before being mounted onto glass slides and coverslipped using Fluoromount-G (Thermo Fisher Scientific).

**Figure 1:**
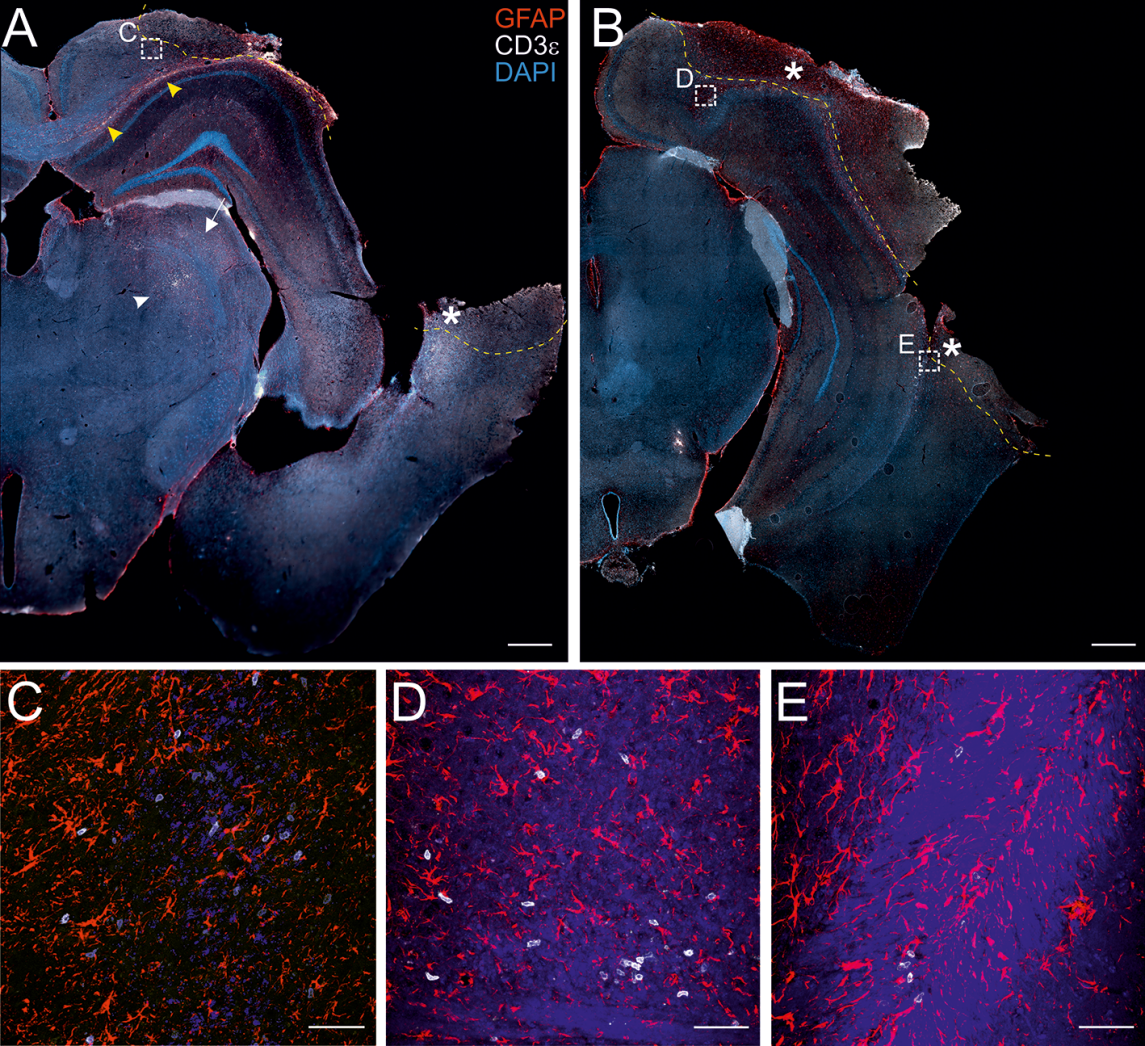
Localization of CD3+ T cells in the perilesional cortices. Representative images of brain sections from CCI mice stained for anti-CD3e (T lymphocytes; white) and anti-GFAP (astrocytes; red), 30 dpi **(A, B)**. The lesion edges in each section are marked with a segmented yellow line. T cells are present within the lesion (star in A and B), in the perilesional cortex (box in A and B, and panel C) and in the corpus callosum (yellow arrow heads in A). CD3+ cells were also observed in the striatum (white arrow in A) and in the thalamus (white arrow head in A). Both scattered cells and presumably-encephalitogenic clusters of T cells (panel **D**) were found within the parenchyma. Panels **(C)** represent a magnification of the area depicted within the white boxes in A. Panels **(D, E)** represent a magnification of the areas depicted within the white box in B. CD3e: white; GFAP: red; DAPI: blue. (A and B, scale bar = 500 µm; C-E, scale bar = 50 µm).

Image acquisition was performed using Zeiss Axio Observer Z1 microscope, equipped with a Zeiss AxioCam MR R3 camera, mounting a 10x lens to obtain images from whole-brain sections. Magnification images of infiltrating T cells (**Figure 1**) were acquired using a Zeiss LSM710 confocal microscope, mounting a 25x LCI plan objective (340 x 340 µm, 21 Z-stack slices/image, 20 µm total thickness).

Image analysis was performed using ImageJ software. ROIs were manually selected on images taken from each stained section. After background subtraction, the mean gray value was measured within each ROI (32).

### Statistical Analysis

#### Data Exclusion Criteria

We conducted eight independent experiments, where a total of n = 16 “WT CCI”; n = 12 “WT naïve”; n = 13 “TG CCI” and n = 10 “TG naïve” mice have been analyzed.

Before statistical analysis, brain-derived samples were checked for their quality, based on total T cell recovery. Each sample has been considered independently, and we evaluated the T cell viability and the total number of T cells recovered. Brain samples where T cell viability was below 75% or the total number of live T cells was below 100 counts were *a priori* excluded from the analyses.

Considering two genotypes (WT and TG) and three experimental conditions (T cells infiltrating the brain tissue ipsilateral to the lesion – “ipsi”; T cells infiltrating the tissue contralateral to the lesion – “contra”, and T cells from naïve brain tissue – “naïve”), a total of n = 12 “WT ipsi”; n = 7 “WT contra”; n = 5 “WT naïve”; and n = 10 “TG ipsi”; n = 7 “TG contra”; n = 9 “TG naïve” were finally used for statistical analyses.

T cell viability >90% was used for the quality requirement of spleen and dcLN samples. Moreover, we excluded spleen samples presenting more than 50% of necrotic tissue (defined as dark red non-perfused area in the spleen). Considering two genotypes (WT and TG) and two experimental conditions (CCI and naïve), a total of n = 13 “WT CCI”; n = 12 “WT naïve”; and n = 11 “TG CCI”, n = 9 “TG naïve” spleens were used for subsequent statistical analyses. Deep cervical lymph nodes have been analyzed in n = 4 “WT CCI” and n = 6 “TG CCI” mice.

#### Statistical Analysis of Brain- and dcLNs-Related Data

Due to the small amount of T lymphocytes in naïve brains, brain samples were fully acquired on the flow cytometer, and for each population we analyzed both the absolute counts and the percentage referred to the respective parent population. Statistic models were applied considering the nature of our data (counts or percentages) and the experimental groups analyzed. A binomial negative regression was applied to assess statistical differences in the counts of total T cells, of CD4+, and of CD8+ cells between the two genotypes or within the same genotype between independent data. The binomial negative regression considered both genotype and treatment and their interaction. Because data from ipsi and contralateral brain sides are dependent within the same genotype, a linear mixed model was used to evaluate the differences in the total number of CD4+ and CD8+ T lymphocytes between “WT ipsi” vs. “WT contra” or “TG ipsi” vs. “TG contra”. As the data were not normally distributed (Shapiro-Wilk test p-value < 0.05), statistical differences between independent data in CD4+ and CD8+ T cell populations (expressed as percentage of T cells), as well as in the percentages of respective subpopulations expressing CD44 and/or CD69 antigens, were analyzed performing the Kruskal Wallis test. Dependent data within the same genotype (ipsi vs. contra) were analyzed performing the paired samples Wilcoxon signed ranked test. In all tests, Bonferroni correction was used to adjust p-values in multiple comparison.

#### Statistical Analysis of Data From Spleen

All data from spleen are expressed as percentage of the parent population. After establishing the normal distribution of the data (as well as skewness and kurtosis by D’Agostino K-squared test), statistical differences were analyzed performing the Kruskal Wallis test or the paired samples Wilcoxon signed ranked test, depending on the nature of the data (independent or dependent), followed by Bonferroni adjustment.

#### Statistical Analysis of Magnetic Resonance Imaging Data

The differences in contusion volume and in brain atrophy were analyzed performing the Kruskal Wallis test (21 dpi) or using a linear mixed model to evaluate the differences between day 3 and day 14 post-TBI. Correlation between TBI-related tissue loss and infarct volume was analyzed by Pearson linear regression, after checking for normal distribution of data as described above.

Statistical analyses were performed using R v3.5.3 software/computing environment (The R foundation for statistical computing). All software packages (MASS, psych, agricolae, multcomp, and lme4) (33–37) were taken from the Comprehensive R Archive Network mirror sites (CRAN; http://CRAN.R-project.org/package=boot). Significance was accepted at the level of p <0.05.

## Results

### T Cells Preferentially Infiltrate the Cortical Areas Ipsilateral to the Lesion

The presence of infiltrating T lymphocytes in the parenchyma is a signature of brain lesion. At a chronic time point after TBI, we localized the T cell presence in the area of injury and in other brain areas not directly affected by the penetrating injury. For this purpose, we stained brain sections of both WT and TG mice at 30 days post-injury (dpi) for the presence of CD3, a specific marker of T lymphocytes. As expected, T cells are present within the boundaries of the injured area (**Figures 1A, B**). CD3+ cells are also spread throughout the cortical parenchyma, both in proximity to the lesion core (**Figure 1C**) and in more distal areas ipsilateral to the lesion along the cortical layers. Positive immunostaining was also found along the corpus callosum (**Figures 1A, B** and magnification in D), the striatum, the hippocampus, and the thalamus ipsilateral to the lesion (**Figure 1A**). Dim CD3+ signal was present in the contralateral hemisphere, indistinguishable from non-injured mice (data not shown). There was no evident difference in T cell distribution between WT and TG mice: unevenly scattered T cells (**Figures 1C, E**) and T cell clusters (**Figure 1D**) were both observed within the parenchyma, in the perilesional areas.

Next, we quantified and characterized the populations of infiltrating T lymphocytes using flow cytometry, focusing on the neo-cortical areas (cortices and hippocampi), excluding the lesion area, which is characterized by a dysregulated entrance of immune cells (38).

**Figure 2:**
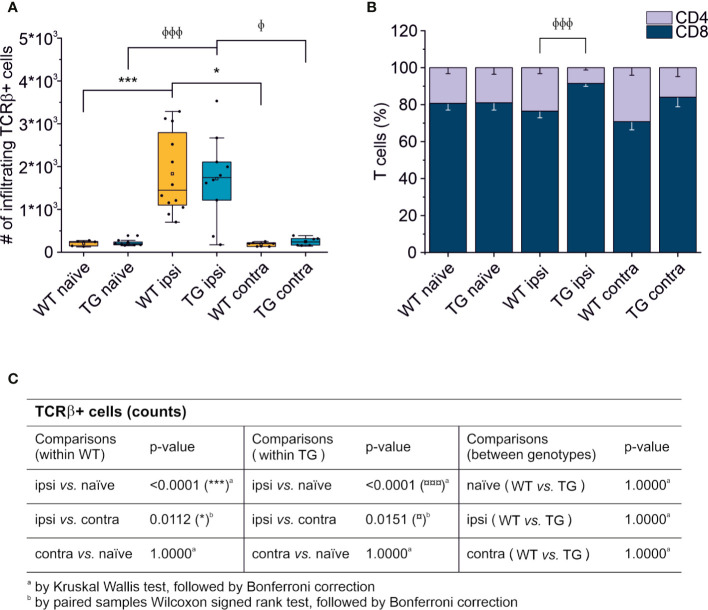
T cell brain infiltration is confined to the perilesional cortices, 30 dpi. Box plot representing the number of infiltrating T cells, defined by expression of TCRβ **(A)** and stacked bargram representing the percentage of CD4+ and CD8+ T cells **(B)** in the brain of WT and TG mice, as analyzed in the perilesional and contralateral cortices (ipsi and contra, respectively; WT ipsi, n = 12; WT contra, n = 7; TG ipsi, n = 10; TG contra, n = 7), or in intact cortices from respective naïve mice (WT naïve, n = 5, TG naïve, n = 9). Independently from the genotype, a significant infiltration of TCRβ+ T cells was observed in the perilesional areas but not in the contralateral hemispheres (comparable to naïve non-injured brains). The majority of brain-infiltrating T cells presented a CD8 phenotype. In the TG CCI mice, there was a significant skew of CD4/CD8 ratio towards CD8+ T cells. Table **(C)** summarizes the results of the statistical analysis in T cell counts between the experimental groups. In **(A)** boxes represent the 25–75% value range, including the median value, indicated with the line. Whiskers represent 1.5x standard deviation (SD). □ indicates the mean value. In the stacked bargram, data are presented as mean ± standard error of the mean (s.e.m.). A binomial negative regression or a linear mixed model was applied to assess statistical differences in the counts of total T cells. The Kruskal Wallis test or the paired samples Wilcoxon signed ranked test was used for the analysis of CD4 and CD8 frequency distribution. ^φ^p < 0.05 and ^φφφ^p < 0.001 vs. TG ipsi. *p < 0.05 and ***p < 0.001 vs. WT ipsi. In all tests, Bonferroni correction was used to adjust p-values in multiple comparisons.

Thirty days after brain trauma induction in TG and littermate WT mice, leukocytes were purified separately from the perilesional and the contralateral cortices (or from the cortex of both WT and TG naïve mice). T cells were identified by staining for T cell receptor (TCRβ) and the presence of the co-receptors CD4 and CD8. Live T cell counts per experimental condition is reported in **Figure 2**. A significant ~10-fold increase of infiltrating T cells was found in both WT (median = 1,449; Q3-Q1 = 1,692) and TG (median = 1,741; Q3-Q1 = 892) mouse brains in the perilesional cortices, compared to corresponding naïve non-injured mice (WT naïve: median = 242; Q3-Q1 = 105; TG naïve: median = 197; Q3-Q1 = 66; for statistical analysis, see **Figure 2C**). In the cortices contralateral to the lesion, the number of TCRβ+ cells did not different from naïve brains (WT contra: median = 201; Q3-Q1 = 84; TG naïve: median = 239; Q3-Q1 = 155; for statistical analysis, see **Figure 2C**). No genotype-related differences were observed (**Figure 2A**).

### Perilesional-Infiltrating T Cells Have a Predominant CD8+ Phenotype, and the Constitutive Lack of mLVs Is Associated with a Depressed CD4-Mediated T Cell Response

**Figure 3:**
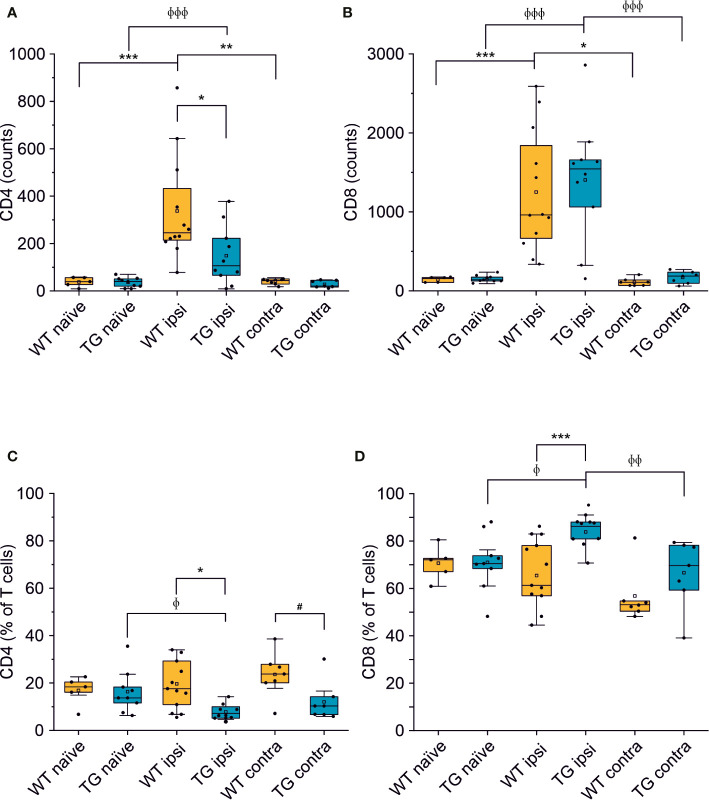
The number of CD4+ but not of CD8+ T cells is reduced in the brain of K14-VEGFR3-Ig mice after TBI. Box plots representing the number and frequency of CD4+ T cells (**A, C**, respectively) and CD8+ T cells (**B, D**, respectively), in the brain of WT and TG mice, as analyzed in the perilesional and contralateral cortices (WT ipsi, n = 12; WT contra, n = 7; TG ipsi, n = 10; TG contra, n = 7), or in intact cortices from naïve mice (WT naïve, n = 5, TG naïve, n = 9). A drastic reduction in the number of CD4+ T cells was found in TG mice after injury. A binomial negative regression or a linear mixed model was applied to assess statistical differences in the counts of CD4+ and CD8+ T cells. The Kruskal Wallis test or the paired samples Wilcoxon signed ranked test was used for the analysis of frequency distribution. *p < 0.05; **p < 0.01 and ***p < 0.001 vs. WT ipsi. ^φ^p < 0.05; ^φφ^p < 0.01 and ^φφφ^p < 0.001 vs. TG ipsi. ^#^p < 0.05 vs. WT contra. In all tests, Bonferroni correction was used to adjust p-values in multiple comparisons.

We next analyzed the CD4:CD8 ratio within the infiltrating T cells (**Figure 2B**) and found a prevalence of CD8+ T cells in all the experimental conditions, regardless of the presence of brain injury. However, limited to the perilesional cortex of TG mice, we detected a significant skew of the CD4:CD8 ratio towards CD8+ cells (CD4:CD8 ratio TG ipsi = 0.097 ± 0.053; WT ipsi = 0.350 ± 0.197; ChiSq: 8.836, mean ranks: 5.50/13.27, p = 8e-04), while the ratio in the contralateral cortex did not differ between the two genotypes (CD4:CD8 ratio TG contra = 0.221 ± 0.247; WT contra = 0.456 ± 0.212; ChiSq: 2.469, mean ranks: 5.43/8.83, p = 0.120). To better understand how the lack of mLVs affects the T cell-mediated neuro-immune response, we analyzed both the absolute numbers of CD4 and CD8 subpopulations and their relative frequency. Data analysis shows a reduction of the total number of CD4+ T cells infiltrating the perilesional cortices of TG (median = 106; Q3-Q1 = 156) compared to WT mice (median = 245; Q3-Q1 = 218; ex. coef.: -0.82, p = 0.033 TG ipsi vs. WT ipsi) (**Figure 3A**). No differences were observed in the absolute number of infiltrating CD8+ T cells between the genotypes (**Figure 3B**). Despite no differences in absolute numbers of both CD4 and CD8 populations in the contralateral cortices of injured WT and TG mice, we found a significant reduction in the frequency of CD4+ T cells in transgenic mice (TG contra = 12.04 ± 8.47%; WT contra = 23.59 ± 9.52% of T cells; ChiSq: 3.931, mean ranks: 5.29/9.71, p = 0.042) and a relative increase in the frequency of CD8+ T cells (**Figures 3C, D**), indicating a specific impairment in the CD4-mediated neuro-immune response. As mLVs are involved in the drainage of solutes from the interstitial and cerebro-spinal fluids mainly to the dcLNs (15, 16), we hypothesized that mLVs absence in TG mice can affect the priming of the evoked neuro-immune response. The analysis of the T cell subpopulations in the dcLNs indeed revealed a marked difference between the two genotypes. As expected, we found a significantly lower number of T cells in the dcLNs of the TG-CCI mice (median = 73,542; Q3-Q1 = 21,342) compared to their WT-CCI littermates (median = 220,434; Q3-Q1 = 88,745; p = 0.006). However, TG-CCI mice had a higher frequency of CD4+ T cells (TG CCI = 63.98 ± 5.67%; WT CCI = 51.40 ± 1.93% of T cells; ChiSq: 6.545, mean ranks: 7.50/2.50, p = 0.0017) (**Supplementary Figure 3**). Within the CD4+ T cell subpopulation in the TG mice, cells have predominantly a CD44^hi^CD69^+^ phenotype, while in the WT mice the predominant population is CD44^int^CD69^-^ (**Supplementary Figure 3**). No differences were found in the frequency of Tregs.

**Figure 4:**
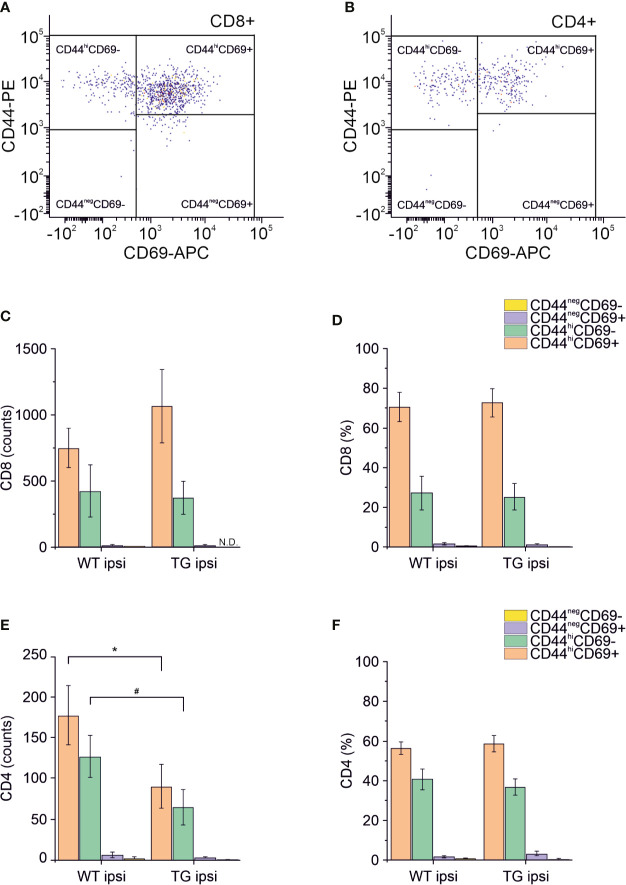
Analysis of CD69 and CD44 T cell activation and memory markers in CD4+ and CD8+ subpopulations. Pseudocolor dot plots **(A, B)** represent gated subpopulations CD69 vs. CD44 of CD8+ and CD4+, respectively. Bargrams in **(C, D)** show respectively the counts and frequencies of CD8+ T cell subpopulations, as analyzed in the perilesional cortices of WT and TG mice. No significant differences in CD8+ subpopulations were found between genotypes. In CD4+ subpopulation, instead, we observed a significant reduction in the counts of CD44^hi^CD69+ and CD44^hi^CD69- subpopulations **(E)**, in K14-VEGFR3-Ig compared to WT mice. However, no differences were observed in the different subpopulation frequencies **(F)**. Data are presented as median ± SD. A binomial negative regression was applied to assess statistical differences in the counts of total T cells between WT ipsi and TG ipsi. The Kruskal Wallis test was used for the analysis of frequency distribution. ^#^p < 0.05; *p < 0.05 vs. WT ipsi.

Different subpopulations of CD8+ and CD4+ T cells exist, with specific and opposing functions: we characterized both the CD8+ and CD4+ subpopulations in the brain for the surface expression of the antigens CD44 (a memory and activation marker) (39, 40) and CD69 (an activation and tissue retention marker) (41). In the perilesional cortex of both WT and TG mice, CD8+ T cells had a predominant CD44^hi^CD69+ phenotype (69.78 ± 22.85% and 72.05 ± 19.95% of CD8+ T cells, in WT ipsi and TG ipsi, respectively) (**Figures 4A, C, D**). In the mouse, the expression of CD69 together with high levels of CD44 define a specific subpopulation of T cells called mature resident memory T cells (T_RM_) (42–44), which are generated and persist in the tissue at the site of a primary infection (43, 45) and provide a first and powerful line of adaptive cellular defense.

The second-highest expressed CD8+ subpopulation (representing 27.07 ± 26.10% in WT and 25.24 ± 18.85% in TG mice) presented a CD44^hi^CD69- phenotype, characteristic of effector memory T cells (43). The presence of other CD8+ subpopulations among perilesional infiltrating T cells was negligible. No genotype-related difference was found (**Figures 4C, D** and **Supplementary Table 2**).

Among CD4+ perilesional infiltrating T cells, we found a similar frequency of CD44 and CD69 expressions, with a slight prevalence of CD44^hi^CD69+ over CD44^hi^CD69- T lymphocytes (**Figures 4B, E, F**) in both genotypes. The overall frequency distribution of the different subpopulations was identical between the two genotypes (**Figures 4E, F** and **Supplementary Table 2**).

### K14-VEGFR3-Ig Mice Present a Different Temporal Profile of the T Cell-Mediated Neuroimmune Response After Traumatic Brain Injury

**Figure 5:**
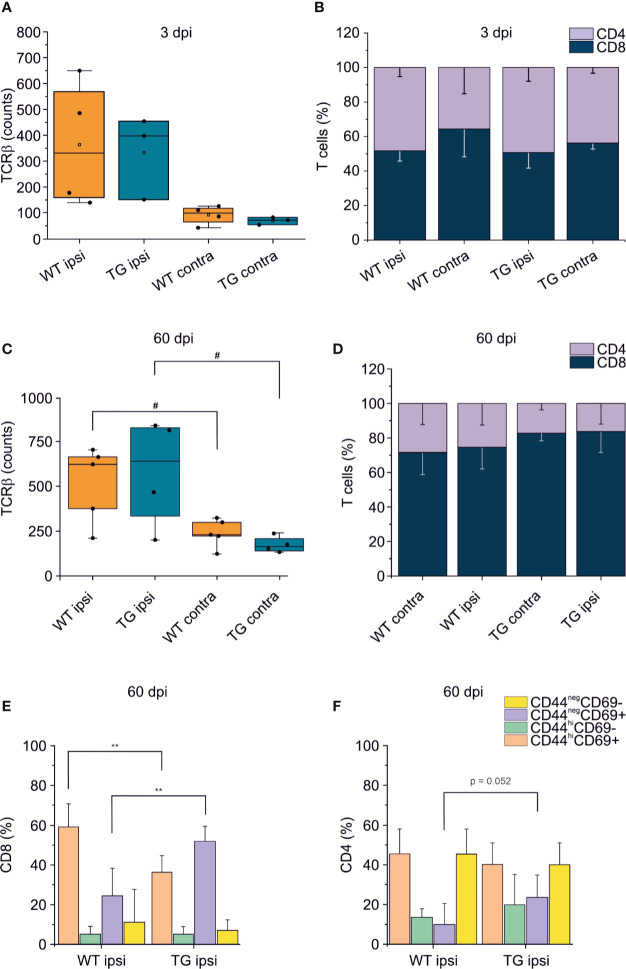
T cell immune response after TBI progress differently in K14-VEGFR3-Ig and WT littermate mice. Panels **(A, B)** represent the number and frequency of TCRβ+ T cells **(A)** and the CD4/CD8 ratio **(B)** in the brain of WT and TG mice, as analyzed in the perilesional and contralateral cortices 3 days post injury (WT ipsi, n = 4; WT contra, n = 4; TG ipsi, n = 3; TG contra, n = 3). No differences between the genotypes have been observed. **(C–F)** Analysis of T cells infiltration in the brain of K14-VEGFR3-Ig and WT littermate mice 60 days post-injury (WT ipsi, n = 5; WT contra, n = 5; TG ipsi, n = 4; TG contra, n = 4). Box plot represents the number of infiltrating T cells, defined by expression of TCRβ **(C)** and stacked bargram represents the percentage of CD4+ and CD8+ T cells **(D)** in the perilesional areas (ipsi) and correspondent contralateral areas (contra) of WT and TG mice. Bargrams in **(C, D)** show respectively the frequencies of CD8+ and CD4+ T cell subpopulations, as analyzed in the perilesional cortices of WT and TG mice. In CD8+ subpopulation we observed a significant reduction in the frequency of the CD44^hi^CD69+ subpopulation in K14-VEGFR3-Ig compared to WT mice, which corresponded to the increase in the frequency of CD44^neg^CD69+ phenotype. In CD4+ subpopulation, instead, we did not observed differences in distribution between the two genotypes. Data are presented as median ± SD. A binomial negative regression or a linear mixed model was applied to assess statistical differences in the counts of TCRβ + T cells. The Kruskal Wallis test was used for the analysis of frequency distribution. **p < 0.01 vs. WT ipsi. #p < 0.05 vs. respective contra. In all tests, Bonferroni correction was used to adjust p-values in multiple comparisons.

To confirm that the elicited neuro-immune response is specifically affected in the K14-VEGFR3-Ig mice, we analyzed in two different cohort of mice the phenotype of the brain-infiltrating T cells at 3 and 60 dpi. As previously reported (7, 9–11, 46), T cell infiltration peaks at 3 dpi: however, this time frame is not compatible with the priming of the adaptive immune response, and the infiltration of T cells is function of the circulating compartment. At this time point, in our experimental preparations, we did not observe a significant increase in the number of TCRβ+ cells infiltrating the perilesional areas in either of the genotypes (WT ipsi median = 331.5, Q3-Q1 = 409; WT contra median = 99, Q3-Q1 = 53.5; TG ipsi median = 397, Q3-Q1 = 302; and TG contra median = 72, Q3-Q1 = 27) (**Figure 5A**). This could be explained by the fact that, at 3 dpi, T cells mainly enter the brain and accumulate in the area of lesion (removed in our preparation), where the blood-brain barrier (BBB) is damaged. Moreover, analysis of the CD4:CD8 ratio within the infiltrating T cells (**Figure 5B**), did not reveal any difference between WT and TG mice (CD4:CD8 ratio TG ipsi = 1.032 ± 0.323; WT ipsi = 0.964 ± 0.198; ChiSq: 0.5, mean ranks: 4.66/3.50, p = 0.530), thus suggesting that T cells at 3 dpi are recruited independently of the mLVs-dcLNs circuit activation. Next, we characterized the T cell infiltration at 60 dpi chronic time point, to evaluate the progression of the neuro-immune response in the two genotypes. Presence of TCRβ+ T cells in both the genotypes was higher in the perilesional areas (WT ipsi median = 625; Q3-Q1 = 291; TG-ipsi median = 642.5; Q3-Q1 = 497.5), compared to the contralateral cortices (WT-contra median = 227; Q3-Q1 = 77, p = 0.014 vs. WT-ipsi; TG-contra median = 163; Q3-Q1 = 69.5, p = 0.062 vs. TG-ipsi). As observed at 30 dpi, we found a prevalence of CD8+ T cells among the infiltrating lymphocytes, however no genotype or lesion effect was observed from the analysis of CD4:CD8 ratio (CD4:CD8 ratio TG ipsi = 0.220 ± 0.184 WT ipsi = 0.379 ± 0.254; TG contra = 0.218 ± 0.058; WT contra = 0.443 ± 0.162). Analyses of CD4+ and CD8+ subpopulations, revealed fundamental changes in subpopulation frequencies between the two genotypes. Among CD8+ T cells, CD44^hi^CD69+ phenotype was prevalent in WT ipsi, while the CD44^neg^CD69+ phenotype was prevalent in TG ipsi (CD44^hi^CD69+: 60.58 ± 11.26% and 34.75 ± 8.00% in WT ipsi and TG ipsi respectively, p = 0.009; CD44^neg^CD69+: 28.53 ± 14.01% and 53.10 ± 7.27% in WT ipsi and TG ipsi respectively, p = 0.004). These data suggest an activation of CD8-mediated neuro-immune response in the perilesional area of K14-VEGFR3-Ig mice, while cytotoxic cells resident in the injured brain of WT mice conserve a memory phenotype. Within CD4+ population, most of the cells in both the genotypes presented a CD44^neg^CD69- phenotype. However, in TG mice we observed a tendency towards a frequency increase in the CD44^neg^CD69+ subpopulation (6.44 ± 11.91% and 23.98 ± 10.90% in WT ipsi and TG ipsi respectively, p = 0.052), supporting the hypothesis of a specific activation of the neuro-immune response in the K14-VEGFR3-Ig mice at 60 dpi.

### Cortical Lesion is Similar in K14-VEGFR3-Ig Mice and in Their Wild Type Littermates

**Figure 6:**
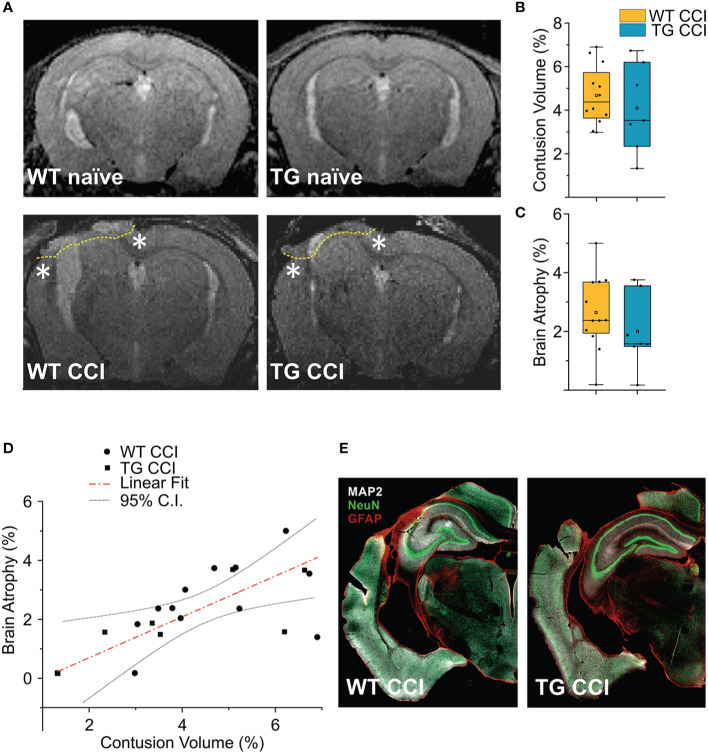
TBI-induced lesions does not differ between the two genotypes, as inferred by the analysis of MRI at 21 dpi. **(A)** Representative MR images of WT naïve, WT CCI, TG naïve and TG CCI brains. Perilesional cortices in WT CCI and TG CCI brains are marked with stars. Box plots in **(B, C)** illustrate the genotype effect on the percentage of contusion volume and of brain atrophy, respectively, over the volume of the hemisphere ipsilateral to the lesion. No significant differences were observed between TG K14-VEGFR3-Ig and WT mice. For the definitions of the contusion volume and of brain atrophy see the main text. **(D)** When considering the contusion volume and the brain atrophy independently from the genotype, we found a direct correlation between the two parameters. **(E)** Representative images of WT CCI and TG CCI brains stained for MAP2, NeuN, and GFAP at 30 dpi. No differences in neuronal damage or in neuroinflammation were visible between the two genotypes, supporting the MRI *in vivo* data. The Kruskal Wallis test was used for the analysis of infarct volume and of tissue loss between the two genotypes. CI: 95% confidence interval. For box plot explanation, refer to the legend of **Figure 2**.

Analyses of MRI images acquired 21 days after TBI induction revealed a T2 intensity increase in the ipsilateral hemisphere. The increase of T2 intensity was observed in parietal-temporal cortices, mainly involving the somatosensory and visual cortices (**Figure 6A**), expanding in a few cases to the underlying hippocampus (**Supplementary Figures 5C, D**). No significant change of T2 intensity was found between the two genotypes. In the WT CCI group the contusion volume was 4.53 ± 1.33%, and 4.09 ± 2.00% in the TG CCI animals (ChiSq: 0.579, mean ranks: 8.71/10.75, p = 0.463) (**Figure 6B**). Relative brain atrophy was 2.42 ± 1.09% in WT CCI mice and 2.00 ± 1.26% in TG CCI mice (ChiSq: 1.400, mean ranks: 8.00/11.17, p = 0.248) (**Figure 6C**). Correlation between contusion volume and relative brain swelling was compared in transformed data analyzed by linear regression. When considering the individual values independent of the genotype, the contusion volume values significantly correlated with the values of relative brain atrophy (r = 0.57; p = 0.023) (**Figure 6D**). No significant correlation was found between the contusion volume and the mean value of the brain atrophy in both the TG CCI group (r = 0.74; p = 0.064), and in the WT CCI mice (r = 0.37; p = 0.331). No differences in lesion progression between WT-CCI and TG-CCI mice have been found from the analysis of T2 MR Images acquired at 3 and 14 days post-injury (**Supplementary Figures 5A, B**).

It must be noted that we have identified the lesion size as the hyper-intense signal in the cortical area observed in the T2 weighted images. Our analysis, albeit clinically relevant, suffers from a lack of spatial definition and is affected mostly by the formation of the cyst at the site of injury (27, 47). Therefore, subtle although significant differences in the lesion size can be underestimated. However, the analysis of MAP-2 staining in the brain of the WT CCI and TG CCI animals confirmed the MRI results and did not show any genotype-related differences (**Figure 6E**).

### K14-VEGFR3-Ig Mice Present a Peripheral Lymphopenia, Which is Exacerbated After Traumatic Brain Injury

**Figure 7:**
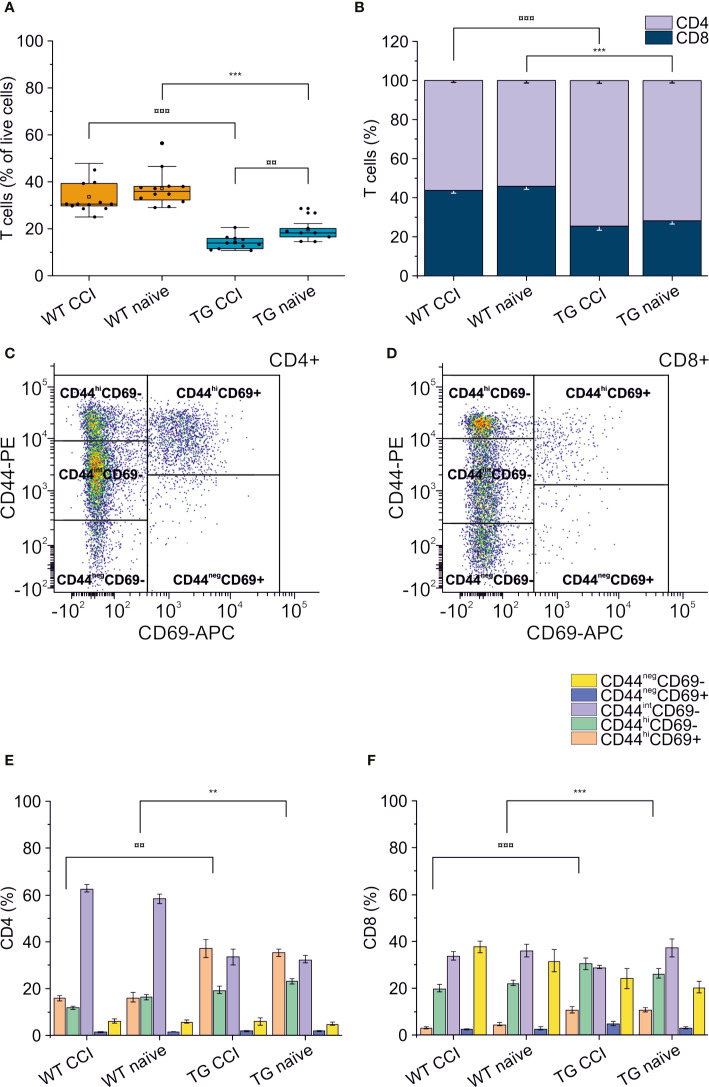
Peripheral immune response in the spleen. The percentages of T cells in the spleen of WT naïve and CCI mice and of TG naïve and CCI mice are presented in the box plot in panel **(A)**. Stacked bargrams in **(B)** represent the relative percentages of CD4 and CD8 in T cell population, in WT and K14-VEGFR3-Ig mice. K14-VEGFR3-Ig mice present a drastic reduction of T cells compared to WT littermates, due to a decrease in CD8+ T cell frequency. **(C, D)** Representative pseudocolor dot plots and gating strategies for CD4+ and CD8+ T cell subpopulation analysis, respectively. Bargrams in **(E, F)** show respectively the frequencies of CD4+ and CD8+ T cell subpopulations, as analyzed in WT and TG mice. Significant differences in the frequencies of both CD4+ and CD8+ subpopulations have been observed. The Kruskal Wallis test or the paired samples Wilcoxon signed ranked test was used for the analysis of frequency distribution. ^¤¤^p < 0.01 and ^¤¤¤^p <0.001 vs. TG CCI. **p < 0.01 and ***p < 0.001 vs. WT naïve. In all tests, Bonferroni correction was used to adjust p-values in multiple comparison. For box plot and stacked bargram explanation, refer to the legend of **Figure 3**.

Alterations of systemic immunity are frequent in TBI patients. We analyzed the levels and the frequency of different T cell subpopulations in the spleen of WT and TG mice, one month after TBI induction. As previously described (48), K14-VEGFR3-Ig mice show a moderate lymphopenia compared to littermate WT mice (percentage of T cells over live cells in WT naïve: 37.26 ± 7.67%; vs. TG naïve: 19.69 ± 4.96%; ChiSq: 14.746, mean ranks: 5.00/15.50, p = 1e-04) (**Figure 7A**). In TG mice, but not in WT mice, we found a significant reduction in the total T cell frequency after TBI (WT CCI: 33.68 ± 6.99%; TG CCI: 14.23 ± 2.87% of live cells; ChiSq: 7.695, mean ranks: 7.18/14.55, p = 0.003 TG CCI vs. TG naïve) (**Figure 7A**), confirming that TG mice present an impaired immune response, which relates to the alterations in the lymphatic system. Contrary to what was observed in the brain, the systemic lymphopenia in the K14-VEGFR3-Ig genotype corresponds to a relative frequency reduction in peripheral CD8+ T cells (TG naïve = 25.75 ± 3.61%; WT naïve = 42.70 ± 4.17% of T cells; ChiSq: 14.727, mean ranks: 5.00/15.50, p = 1e-04) (**Figure 7B**). Analysis of the activation markers show a different expression in both CD4+ (**Figures 7C, E**) and CD8+ (**Figures 7D, F**) subpopulations between WT and TG mice, which is trauma independent. Both TG naïve and TG CCI mice, indeed, showed an increased frequency of memory T cells (CD4+CD44^hi^CD69+, CD4+CD44^hi^CD69- and CD8+CD44^hi^CD69+, CD8+CD44^hi^CD69-; for statistical analysis, see **Supplementary Table 3**).

## Discussion

This study analyzes the progression of T cell-mediated neuro-immune response as a result of a single moderate TBI, in a mouse model characterized by a developmental deficiency in the CNS lymphatic system.

Mounting evidence implicates a sustained modulation of T lymphocyte-mediated immune response following TBI, both in patients (49–51) and in animal models of brain injuries (7, 9–11, 46).

A recent publication from Daglas and colleagues characterized for the first time the T cell-mediated immune response in a chronic animal model of TBI, highlighting the role of cytotoxic CD8+ T cells in the progression of TBI pathology (12).

Our data confirm the previous findings, showing a sustained accumulation of CD8+ T lymphocytes, restricted to the non-damaged cortical areas surrounding the lesion and to the underlying corpus callosum, already at 30 dpi (*i.e.*, the early chronic phases after TBI). Moreover, we expand the current knowledge characterizing the phenotype of the accumulating lymphocytes as putative resident memory T cells. Our data suggest a direct in-situ activation of the T cell-mediated immune response, which could play a role in the progression of TBI pathology, as previously indicated (12).

We also found that the congenital lack of the meningeal lymphatic system affects the polarization of the TBI-elicited T cell immune response, and its progression over time. Finally, we found that the adaptive neuro-immune response is prompted even in the absence of a systemic immune reaction.

Specifically, our findings suggest that at early chronic time points after TBI: 1) the immune response in the brain is principally mediated by putative T_RM_ CD8+ cells; 2) the CNS lymphatic system modulates the specific neuro-immune response; 3) the systemic T lymphocyte response does not correlate with the neuro-immunological state of the brain.

Brain trauma results in two phases of tissue injury. The primary injury which is a direct result of the mechanical impact to the brain, is characterized by the activation of the innate immune response and the release of excitotoxic agents. During this acute phase, a massive and dysregulated brain-infiltration of T cells has been reported (52, 53). This infiltration is presumably confined to the area of the lesion, since we observed a limited number of infiltrating T cells in the perilesional non-injured areas, 3 days after TBI induction (**Figure 5A**). A secondary tissue damage, resulting in a diffuse and long-lasting injury, usually develops after months/years from the primary injury (54–56). This is characterized by additional neurodegeneration developing independently from the mechanical trauma and by the formation of a fibrotic scar tissue in the injured area (57) (**Figure 6E**). It has been recently suggested that the development of secondary injuries is sustained by activated memory CD8+ T cells (12). In CCI mouse model (similar to the one used in this study), the authors observed that the modulation of the cytotoxic lymphocytes resulted in the reduction of the lesion size and in the improvement of the neurological outcomes analyzed 32 weeks after injury.

In similar experimental conditions, we observed that CD8+ T lymphocytes with a CD44^hi^CD69+ phenotype are already present in the perilesional areas (but not in the correspondent contralateral cortices) one month after TBI. Since CD69 is an early marker of T cell activation (41) and inhibits tissue egression (45), our data suggest a localized activation of the resident memory CD8+ subpopulation (42–44) restricted to the areas surrounding the primary lesion. In the case of TBI, CD44^hi^CD69+ T_RM_ cells may represent the population designated to defend the non-injured brain from possible infective agents penetrating through the lesion. However, within the chronic neuro-inflammatory environment observed in the perilesional areas (**Figure 6E**), we propose that T_RM_ can activate in a dysregulated way. Indeed, our data indicate that, 2 months after TBI, CD8+ T cells present around the lesion shift towards a CD44^neg^CD69+ phenotype, typical of functional differentiated tissue-resident T cells. This may contribute to the cytotoxic immune response, which characterizes the chronic phases of TBI pathology. Our hypothesis is supported by the data reported by Daglas and colleagues (12), indicating that brain infiltrating CD8+ T cells express and release effector cytokines (Granzyme B and IFNγ). Further studies are required to determine if this adaptive response is antigen specific, and if secondary lesions are the result of an autoimmune-like sequelae of events.

Neuro-immune responses are mainly elicited in the dc- and scLNs (18–20, 58, 59), which are the main receivers of the mLVs. Therefore, the meningeal lymphatics represent an integrated component in the neuro-immune response (15), and we hypothesize that mLV functional impairment can affect the priming of the T cell-mediated neuro-immune response following TBI.

We addressed this hypothesis by inducing TBI in a transgenic mouse, modelling a congenital lymphedema. K14-VEGFR3-Ig mice, expressing soluble VEGFR-3-Ig (21), present alterations in the development of the lymphatic system, resulting in defective growth of mLVs and in sclerotic dcLNs (16, 17). This phenotype has been confirmed in our experimental animals.

We found that the neuro-immune response in the K14-VEGFR3-Ig mice significantly differs from the response observed in WT mice after TBI, suggesting that a developmental defect in the CNS lymphatic system directly affects the CNS regional immune regulation and modulates its chronic activation in the TBI pathology. This hypothesis is supported by the observation that the initial BBB damage-associated T cell infiltration in the perilesional areas was similar in the two genotypes (**Figures 5A, B**), whereas at 30 dpi (and partially at 60 dpi) we found a marked decrease in the CD4+ T cell frequency in the TG mice (**Figures 2A, B** and **Figures 5C, D**). This results in the polarization of the neuro-immune response towards CD8+ cytotoxicity, possibly aggravating TBI outcomes as recently suggested (12). Moreover, at the most chronic time point analyzed in this study (60 dpi), we also observed a different evolution of the CD8-mediated response, with T cells from the brain of TG mice shifted towards a CD44^neg^CD69+ phenotype, and the one from WT littermates still presenting mainly a T_RM_ phenotype (**Figure 5E**). It is important to note, however, that K14-VEGFR3-Ig mice have a compromised peripheral immune response (22, 48, 60, 61), which could affect the local immune response observed in the brain.

Indeed, in chronic TBI animals, the analysis of the T cell subpopulation in the CNS-draining dcLNs also showed a marked difference between the two genotypes. CD4+CD44^hi^CD69^+^ T cells were the predominant subpopulation in TG mice, and CD4+CD44^int^CD69^neg^ T cells were predominant in WT mice (**Supplementary Figures 3D, E**). It has been suggested that CD4+CD44^int^ T cells could represent the fraction of central memory T helper cells expressing IFN-γ, while CD4+CD44^hi^ would preferably be effector memory cells with a Th17 phenotype (62, 63). A polarized Th1/Th17 response has been reported in CNS autoimmune diseases (64) and can enhance the cytotoxicity of CD8+ T cells (7, 12). This would support the differences in the neuro-immune response observed in our TG animals and partially explain the direct correlation we found in these mice between the frequency of CD4+ T cells and the brain atrophy (**Supplementary Figure 3**). However, the panel of antibodies we used for T cell characterization does not allow us to distinguish between the different CD4+ T helper populations (*i.e.*, Th1, Th2, or Th17) without speculation.

Our data suggest that the developmental impairment of mLVs observed in K14-VEGFR3-Ig mice is associated with a different modulation of the adaptive neuro-immunity in response to TBI. We here conjecture on the possibility that in both WT and K14-VEGFR3-Ig mice, as the result of trauma, the brain-derived antigens escape directly into the blood, activating a CD8-mediated immune response in secondary lymphoid organs, with T cells freely accessing the lesion site due to the damage in the BBB. This results in a similar activation of the primary adaptive immune response which eventually generates the brain resident memory T cells. In WT mice, however, our hypothesis is that antigens are partially drained through the mLVs to the dcLNs, eliciting a Th2-mediated response as previously proposed (18, 20). In K14-VEGFR3-Ig mice, where the functional mLVs-dcLNs connection is absent, this specific response however is restrained, as suggested by our data.

Other mechanisms linked to mLV dysfunction can contribute to the modulation of the neuro-immune response. For instance, lymphatic vessels play a direct role in the maturation of T cells (65, 66), and dysfunction of the lymphatics leads to the persistence of immune cells and mediators in tissues, resulting in a chronic inflammation and tissue damage (67). Moreover, recent papers reported that the VEGFR-3 signaling, promoting lymphangiogenesis, is also important to both initiate the acute innate and adaptive immune responses and to regulate the chronic T cell-mediated response (by changing the Treg/Th2 balance), suggesting an immunomodulatory role for this signaling (68, 69).

It is conceivable, therefore, that the inhibition of the VEGFR-3 signaling in K14-VEGFR3-Ig TG mice, and their congenital lack of mLVs, can affect both the type of the elicited neuro-immune response and its progression.

Interestingly, in a recent paper it has been demonstrated that TBI leads to the temporary impairment in meningeal lymphatic drainage, by increasing intracranial pressure (70). These data suggest that, independently from the pre-existing mLV deficit, drainage of the antigens to the dcLNs should be inhibited during the acute phases following brain trauma. In addition, the same authors demonstrated that prior lymphatic defects are related to the increase in the TBI-induced innate and adaptive immune responses (enrichment gene analysis), and to a more pronounced cognitive deficit, when acutely tested after brain injury (3 dpi) (70). However, in their work Bolte and colleagues used the photosensitizer verteporfin to induce the photodynamic ablation of mLVs, a treatment well known to induce the release of free radicals and the increase of local inflammation after verteporfin activation. Therefore, although these recent observations, together with our data, suggest that pre-existing mLV conditions can promote the neuro-immune response and worsen TBI pathology, more unbiased studies need to be provided to confirm this hypothesis.

Our data, indicating a role of T_RM_ cells in the TBI pathology, could also have important clinical implications. TBI patients generally present a delayed secondary immunodeficiency (CNS injury-induced immunodepression, CIDS) (71, 72), which is accompanied by an increased susceptibility to systemic infections and is associated with declining neurological outcome and increased mortality.

Analysis of our data suggest that neuro-immune reaction can be elicited in the CNS even in the presence of a systemic congenital lymphopenia (as observed in K14-VEGFR3-Ig mice), excluding a correlation between the extent of brain infiltration and the level of T cells in the periphery (**Supplementary Figure 4C**). This observation suggests that patients with CIDS could at the same time present a sustained adaptive immune response localized in the brain. Immunomodulatory therapies directly targeting the brain-resident memory T cells could benefit TBI patients without affecting their already compromised systemic immune system.

Therapeutic approaches aimed at downregulating the adaptive immune response after TBI have been tested before (73) with no improvement on the neurological outcome, leading to the hypothesis that the adaptive immune response after brain injuries can have a beneficial activity (74, 75). However, it is important to note that these studies focused on the manipulation of the early wave of T cell infiltration after TBI. Our findings, together with recently published data, indicate that the chronic immune response is the target for the development of specific therapies for the treatment of TBI patients. This includes modulating the progression of the secondary injuries and opening the way to new studies in this direction.

## Limitation of The Study

This work represents a proof of concept for the involvement of adaptive neuro-immunity in TBI pathology and for the role of mLVs in modulating this response.

We are aware that this study presents several limitations and further studies are needed to understand how mLVs regulates the kinetics of activation and brain recruitment of CD8+ T cells after TBI, and the specific role of these cells in the progression of the pathology. A major limitation stems from the use of K14-VEGFR3-Ig mice with a congenital and global deficiency in the mLVs. This results in a compromised peripheral immune response, as previously demonstrated (22, 48, 60, 61) and confirmed by our spleen data. In their paper, however, Thomas and colleagues reported a delayed but robust CD8-mediated response to peripheral immunization and impaired tolerance. In a similar fashion, we have found an increase in the CD8+ T cell response to putative brain-derived antigens. These data confirm the contribution of lymphatic vessels in the modulation of the adaptive immune response and support the hypothesis that the elicited cytotoxic response can escape the intrinsic brain tolerance. Nevertheless, this hypothesis needs to be confirmed in different models that would study the effects of local partial depletion of the mLVs on the activation of the neuro-immune response (e.g., ligation of the dcLNs at the moment of brain injury).

Another limitation of our study is the lack of difference in lesion size between K14-VEGFR3-Ig mice and their WT littermates despite the increase in the number of cytotoxic T cells. As discussed previously, this could be due to limitations in our analytical approach. However, it is also possible that although triggered by cytotoxic T cells, secondary neurodegeneration and associated behavioral correlates may appear at a later time point than the one analyzed in this study. Specific analyses should be conducted in the K14-VEGFR3-Ig mice (and other models of meningeal lymphatic depletion) to assess the long-term effects of mLV deficits on the progression of TBI pathology.

Finally, our analyses focused on TCRb+ T cells, which represent the main population of T cells responsible for the adaptive immune response. Other immune cells (not analyzed in our study) could play an important role in TBI pathology, representing a possible target for future immunomodulatory strategies. Further studies are needed to fully characterize the contribution of the humoral and cellular neuro-immune response in TBI pathology.

## Conclusions

Our study investigated the phenotype of T lymphocytes infiltrating and persisting in the brain after TBI, pointing to the activation of the CD8+ resident memory T cells in the early chronic response. Our findings also support the importance of mLVs and dcLNs in maintaining brain immuno tolerance. We, therefore, propose that the modulation of the neuro-immune response *via* the CNS-lymphatic system, or by directly targeting the brain-resident memory T cells, could offer therapeutic strategies for the treatment of TBI patients.

## Data Availability Statement

The raw data supporting the conclusions of this article will be made available by the authors, without undue reservation.

## Ethics Statement

The animal study was reviewed and approved by the Animal Ethics Committee of the Provincial Government of Southern Finland.

## Author Contributions

SW contributed to the methodology, investigation, validation, data curation, writing, reviewing, and editing. AV contributed to the investigation, data curation, and formal analysis. MV contributed to the investigation, data curation, and formal analysis. BG provided the software and contributed to the formal analysis. MK contributed to the investigation, writing, reviewing, and editing, ER conducted the formal analysis. SA provided the resources and wrote, reviewed, and edited the manuscript. JK supervised the study and acquired the funding. FN conceptualized the study, contributed to the methodology, validated the study, and contributed to the writing, supervision, and funding acquisition. All authors contributed to the article and approved the submitted version.

## Funding

This study has been supported by the Academy of Finland (Academy of Finland research Fellowship #309479/2017—FN), by the Jane and Aatos Erkko Foundation (SA and JK), by the Finnish Brain Foundation Terva Program (SA), and by European Research Council (ERC) under the European Union’s Horizon 2020 research and innovation programme under grant agreement #743155 (SA).

## Conflict of Interest

The authors declare that the research was conducted in the absence of any commercial or financial relationships that could be construed as a potential conflict of interest.
