# Supplementary material for: Developmental Dysfunction of the Central Nervous System Lymphatics Modulates the Adaptive Neuro-Immune Response in the Perilesional Cortex in a Mouse Model of Traumatic Brain Injury
Source: Front Immunol. 2021 Jan 27;11:559810. doi: 10.3389/fimmu.2020.559810 (PMC7873607; doi:10.3389/fimmu.2020.559810)
Supplement: Supplementary file 1 [file DataSheet_1.pdf]

Supplementary Table 1

Genotype	Age (days)	Weight (g)	Anesthesia (isoflurane % during mantainance)	Surgery duration (min)	Anesthesia (isoflurane total volume administered; mL)	Impact velocity (m/s)	Injury (EDH/SDH/SAH)
**C57Bl/6JOla**	222 ± 50	34.7 ± 3.4	1.2 ± 0.0	36 ± 2	2.04 ± 0.06	5.01 ± 0.01	4/4/4
**K14-VEGFR3-Ig**	203 ± 39	36.2 ± 3.3	1.0 ± 0.1	39 ± 4	2.10 ± 0.08	5.01 ± 0.01	1/5/5

Supplementary Table 1: ***Variables related to TBI-induction procedures.*** Standardization of procedures resulted in no statistical differences in the related variables between WT (C57Bl/6OlaJ) and TG (K14-VEGFR3-Ig) mice. Data are relative to the animals considered for the final analyses (spleen - see main text for details). The total volume of isoflurane administered during the surgery refers to the ”induction” and ”mantainance” phases. Epidural hematoma (EDH), subdural hematoma (SDH) and subarachnoid hemorrhage (SAH) were evaluated immediately after TBI induction by visual inspection of the injury area, by a trained researcher.

Supplementary Table 2

BRAIN

	ChiSq	Mean Ranks	p value	ChiSq	Mean Ranks	p value
**CD8+CD44^{hi}CD69+**	*TG ipsi*			*WT contra*		
WT ipsi	0.1263	10.00/9.10	0.7338	---	4.60/9.70	0.040 (*)
TG contra	---	7.14/8.75	0.5400	0.7978	7.28/5.40	0.3973
**CD8+CD44^{hi}CD69-**	*TG ipsi*			*WT contra*		
WT ipsi	0.032	9.75/9.30	0.8652	---	11.40/6.30	0.040 (*)
TG contra	---	8.86/7.25	0.5400	0.7978	5.71/7.60	0.3973
**CD8+CD44^{neg}CD69+**	*TG ipsi*			*WT contra*		
WT ipsi	0.0079	9.37/9.60	0.9324	---	4.80/9.60	0.053
TG contra	---	7.57/8.37	0.7800	2.5592	7.86/4.60	0.1123
**CD8+CD44^{neg}CD69-**	*TG ipsi*			*WT contra*		
WT ipsi	5.779	6.12/12.20	0.0101(**)	---	6.70/8.65	0.460
TG contra	---	6.00/9.75	0.090	0.5566	6.00/7.20	0.4821

	ChiSq	Mean Ranks	p value	ChiSq	Mean Ranks	p value
**CD4+CD44^{hi}CD69+**	*TG ipsi*			*WT contra*		
WT ipsi	0.109	10.50/9.63	0.7514	---	7.50/9.81	0.400
TG contra	---	8.00/8.00	1.000	1.6530	8.28/5.50	0.2118
**CD4+CD44^{hi}CD69-**	*TG ipsi*			*WT contra*		
WT ipsi	0.197	8.87/10.00	0.6704	---	11.19/16.64	0.56
TG contra	---	7.57/8.37	0.780	1.3061	5.85/8.33	0.271
**CD4+CD44^{neg}CD69+**	*TG ipsi*			*WT contra*		
WT ipsi	1.254	11.69/8.77	0.275	---	8.25/9.40	0.680
TG contra	---	5.92/9.81	0.094	0.325	6.50/7.58	0.5911
**CD4+CD44^{neg}CD69-**	*TG ipsi*			*WT contra*		
WT ipsi	0.441	11.00/9.27	0.5222	---	11.00/7.90	0.240
TG contra	---	8.00/8.00	1.000	0.2032	6.57/7.50	0.6718

Supplementary Table 2: Statistical analyses of the CD8+ and CD4+ T cell subpopulation frequencies was performed using the Kruskal Wallis test or the paired samples Wilcoxon signed ranked test. Bonferroni correction was used for multiple analyses (refer to main text for detailed information). * $p < 0.05$; ** $p > 0.01$ by paired samples Wilcoxon signed ranked test.

Supplementary Table 3

**SPLEEN**	**ChiSq**	**Mean Ranks**	**p value**	**ChiSq**	**Mean Ranks**	**p value**
**CD8+CD44^{hi} CD69+**	*TG CCI*			*WT naïve*		
WT CCI	12.631	14.50/5.50	0.0004 (***)	2.719	9.00/13.58	0.1000
TG naïve	0.521	9.94/8.17	0.4881	14.737	17.00/6.50	0.0001(***)
**CD8+CD44^{hi} CD69-**	*TG CCI*			*WT naïve*		
WT CCI	8.347	13.56/6.25	0.0012(**)	1.828	9.45/13.21	0.1825
TG naïve	1.689	10.69/7.50	0.2031	2.020	13.22/9.33	0.1603
**CD8+CD44^{int} CD69-**	*TG CCI*			*WT naïve*		
WT CCI	3.321	6.94/11.55	0.0663	1.045	9.95/12.79	0.3183
TG naïve	3.000	6.75/11.00	0.0825	0.323	1.89/10.33	0.5829
**CD8+CD44^{neg} CD69+**	*TG CCI*			*WT naïve*		
WT CCI	5.755	12.87/6.80	0.0113(*)	0.004	11.40/11.58	0.9493
TG naïve	2.370	11.00/7.22	0.1271	1.459	12.89/9.58	0.2363
**CD8+CD44^{neg} CD69-**	*TG CCI*			*WT naïve*		
WT CCI	7.595	6.25/12.60	0.0024(**)	1.655	13.45/9.87	0.2057
TG naïve	0.231	8.37/9.56	0.6456	7.293	6.78/14.17	0.0037(**)

	**ChiSq**	**Mean Ranks**	**p value**	**ChiSq**	**Mean Ranks**	**p value**
**CD4+CD44^{hi} CD69+**	*TG CCI*			*WT naïve*		
WT CCI	12.008	14.37/5.60	0.0005(***)	0.353	12.40/10.75	0.5655
TG naïve	0.454	9.87/8.22	0.5182	14.727	17.00/5.50	0.0001(***)
**CD4+CD44^{hi} CD69-**	*TG CCI*			*WT naïve*		
WT CCI	11.400	14.25/5.70	0.0007(***)	6.787	7.55/14.79	0.0058(**)
TG naïve	4.083	6.37/11.33	0.0386(*)	10.227	16.00/7.25	0.0003(***)
**CD4+CD44^{int} CD69-**	*TG CCI*			*WT naïve*		
WT CCI	12.631	4.50/13.50	0.0003(***)	1.332	13.25/10.04	0.2581
TG naïve	0.148	9.50/8.55	0.7133	14.727	5.00/15.50	0.0001(***)
**CD4+CD44^{neg} CD69+**	*TG CCI*			*WT naïve*		
WT CCI	5.337	12.75/6.90	0.0156(*)	0.480	10.45/12.37	0.5018
TG naïve	0.592	10.00/8.11	0.4593	2.913	13.66/9.00	0.0878
**CD4+CD44^{neg} CD69-**	*TG CCI*			*WT naïve*		
WT CCI	0.197	8.87/10.00	0.6704	0.000	11.5/11.5	1.0000
TG naïve	0.453	8.12/9.78	0.5182	0.409	10.00/11.75	0.5363

Supplementary Table 3: Statistical analyses of the CD8+ and CD4+ T cell subpopulation in the spleen was performed using Kruskal Wallis test with Bonferroni correction for multiple analyses (refer to main text for detailed information). * $p < 0.05$; ** $p < 0.01$; *** $p < 0.001$

Supplementary Figure 1

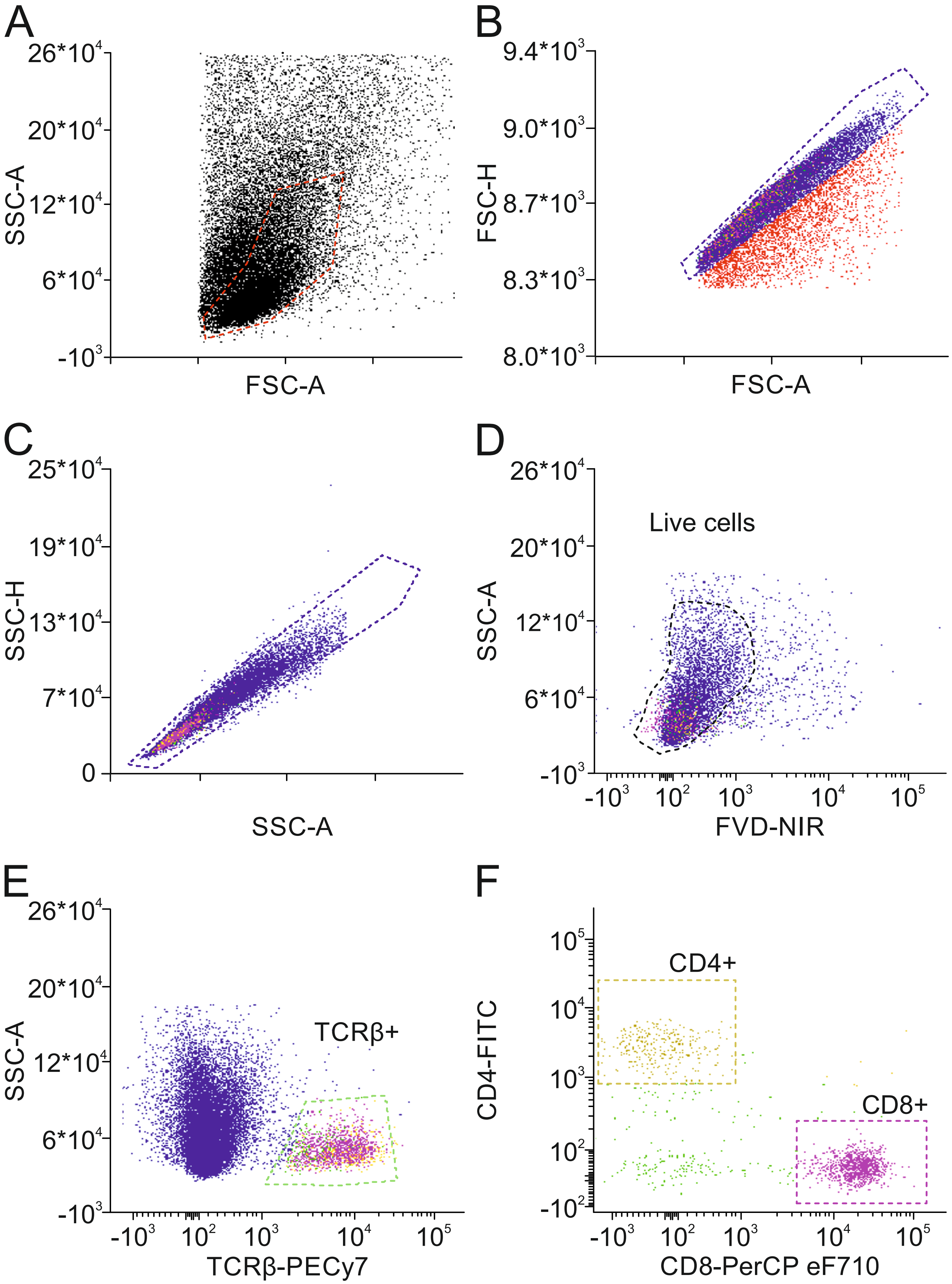

Supplementary Figure 1: Flow cytometry gating strategy for live cell analysis. Mononuclear cells were discriminated out from debris (red dotted line) by light scattering properties in a 2D plot showing forward (FSC-A) *vs.* side scatter (SSC-A) (A). Gated cells were analyzed further with Height (-H) and Area (-A) parameters of FSC and SSC to remove cell doublets (B, C). From these single gated events, live cells were defined as negative for near infrared fluorochrome-conjugated fixable viability dye (FVD-NIR) (D). From live cells, T cells were identified as positive for TCRβ (E), and the lymphocyte subsets were characterized by the expression of CD4 and CD8 cell surface markers (F). Gating was determined using FMOs (see Methods section).

Supplementary Figure 2

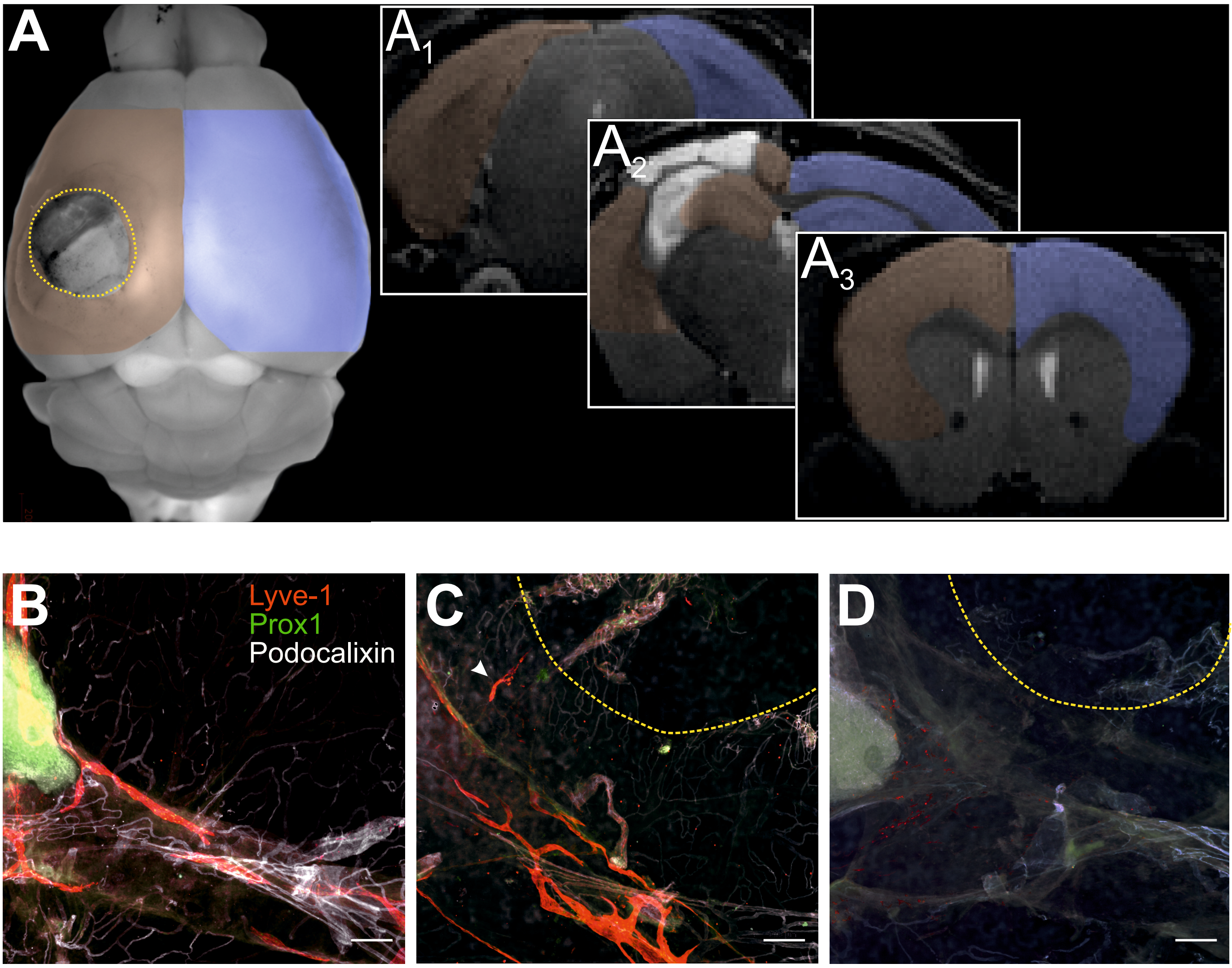

Supplementary Figure 2: Representative macrophotograph **(A)** and MR images **($A_{1\text{-}3}$)** of injured brain. Colored ROIs correspond to the perilesional (orange) and contralateral (blue) areas analyzed in the study. Panels A_1 and A_3 represent respectively the first caudal and the last rostral levels included in the study. Lesion area (delimited by the yellow dotted line in panel A) is clearly identifiable: to avoid peripheral T lymphocyte contamination, tissue from the lesion area was carefully excised and not used for further analyses. **(B-D)** Lymphatic vessels stained in the top skull meninges, in proximity of the lesion site (yellow dotted line in panels C and D). Meningeal lymphatics have been identified along the sinuses in isolated dura preparation from WT-naive (B) and WT-CCI (C) mice. mLVs sprouting towards the craniotomy (white arrowhead in C) has been observed in few WT-CCI mice. No Lyve-1 (red) or Prox1(green dots) lymphatics have been observed in TG-CCI mice (D). Lyve-1: red; Prox1: green; Podocalyxin: white. Scale bars: 200 μm

Supplementary Figure 3

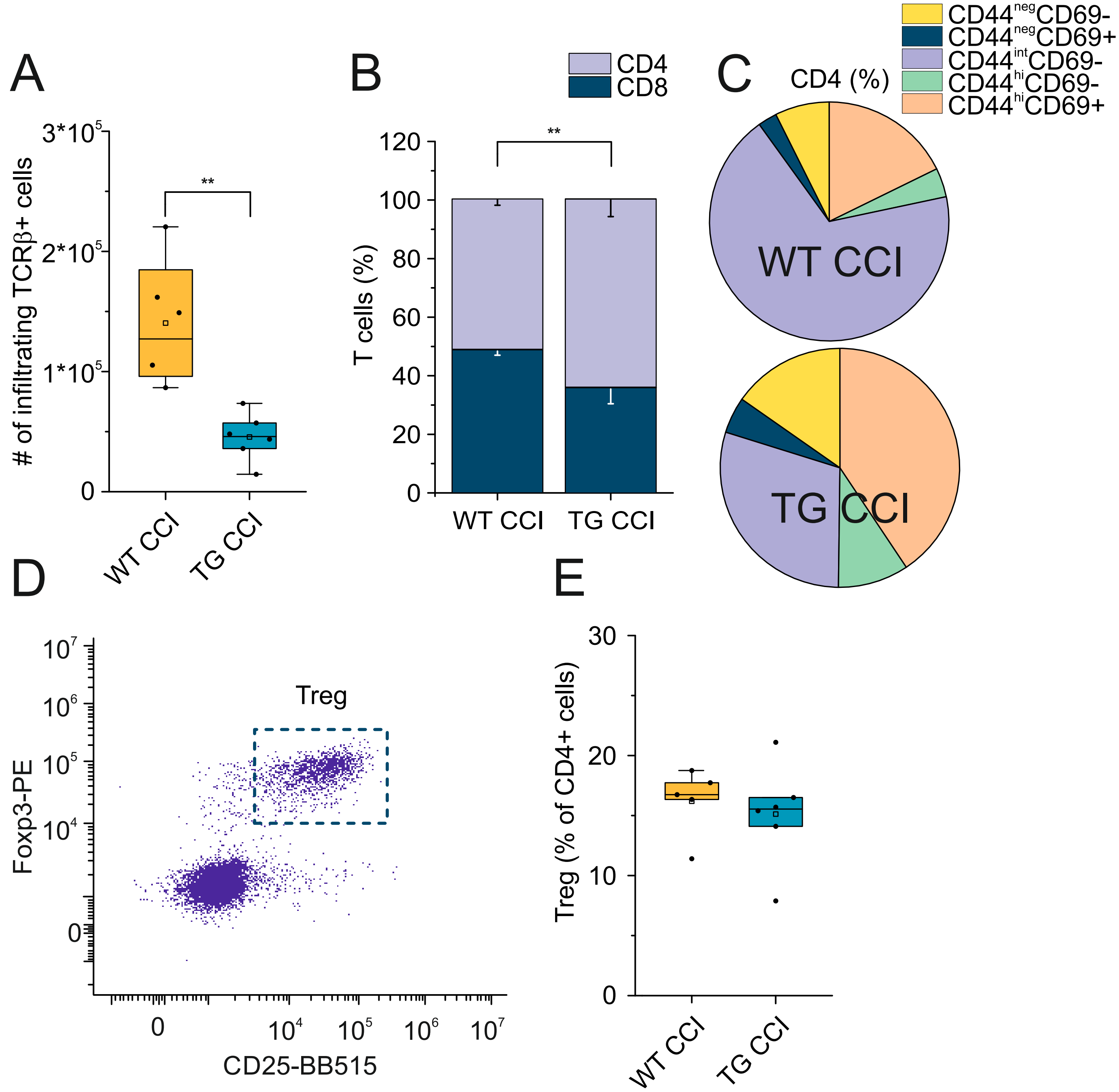

Supplementary Figure 3: ***Analysis of T cell populations in the dcLNs, 30 dpi -*** Box plot in (A) and stacked bargram in (B) represent respectively the total number and the CD4:CD8 ratio of T cells in the dcLNs (left and right side combined for each mouse). Pie charts in (C) represent the frequencies of CD4+ T cell subpopulations. (D) Representative dot plot and gating strategy for the analysis of the CD4+FoxP3+CD25+ Treg cell subpopulation. (E) Box plot representing the percentages of Tregs in the dcLNs of WT CCI (n = 5) and TG CCI (n = 6) mice. ** p < 0.01 WT-CCI *vs.* TG-CCI

Supplementary Figure 4

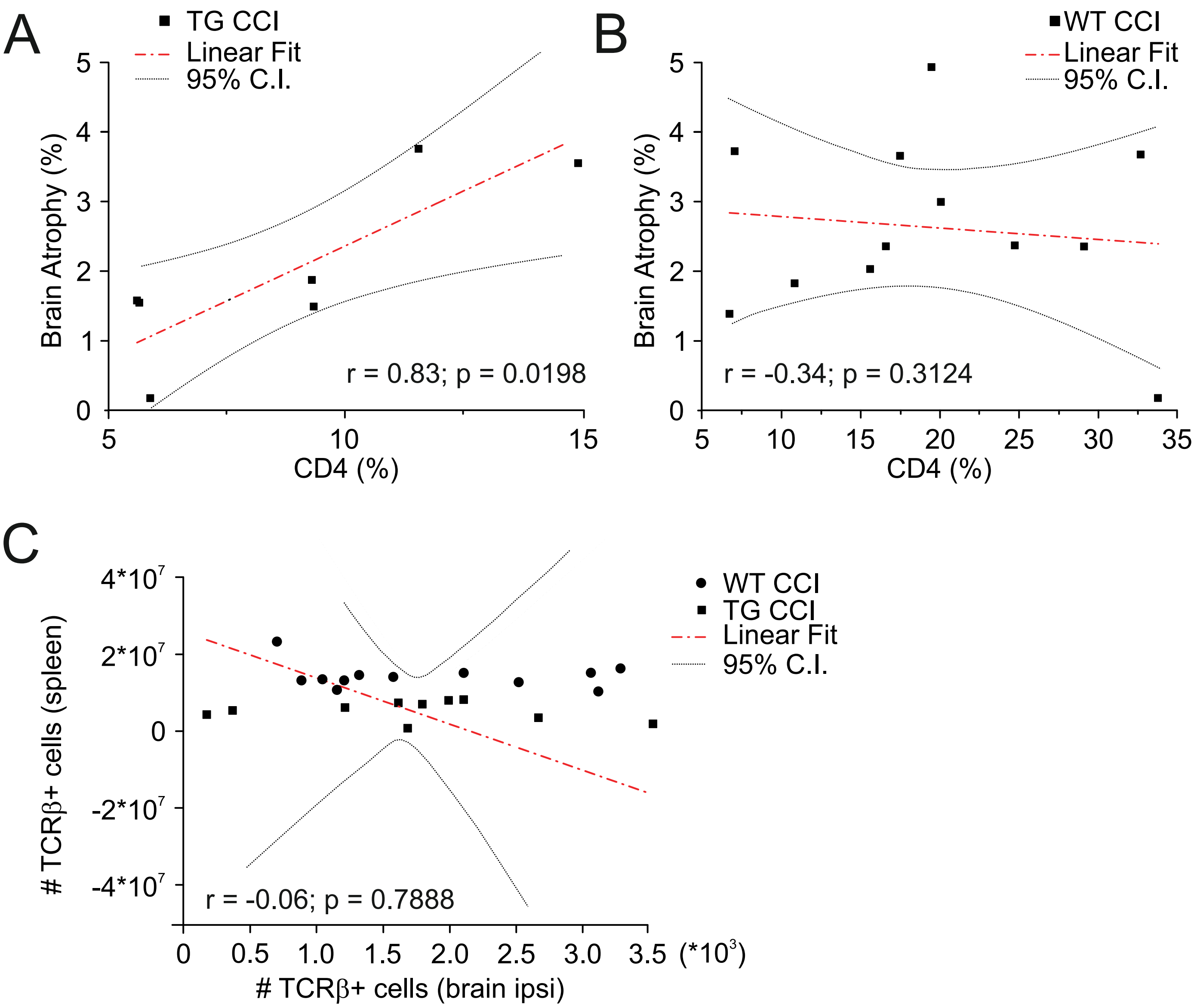

Supplementary Figure 4: CD4+ T cell frequency directly correlate with the percentage of tissue loss in TG CCI (n = 7) **(A)** but not in WT CCI mice (n = 12) **(B)**. Scatter plot in **(C)** show the correlation between the calculated total number of T cells in the spleen and the number of infiltrating T cells in the perilesional cortex in each analyzed mouse (WT CCI, n = 12; TG CCI, n = 10). Pearson's linear regression.

Supplementary Figure 5

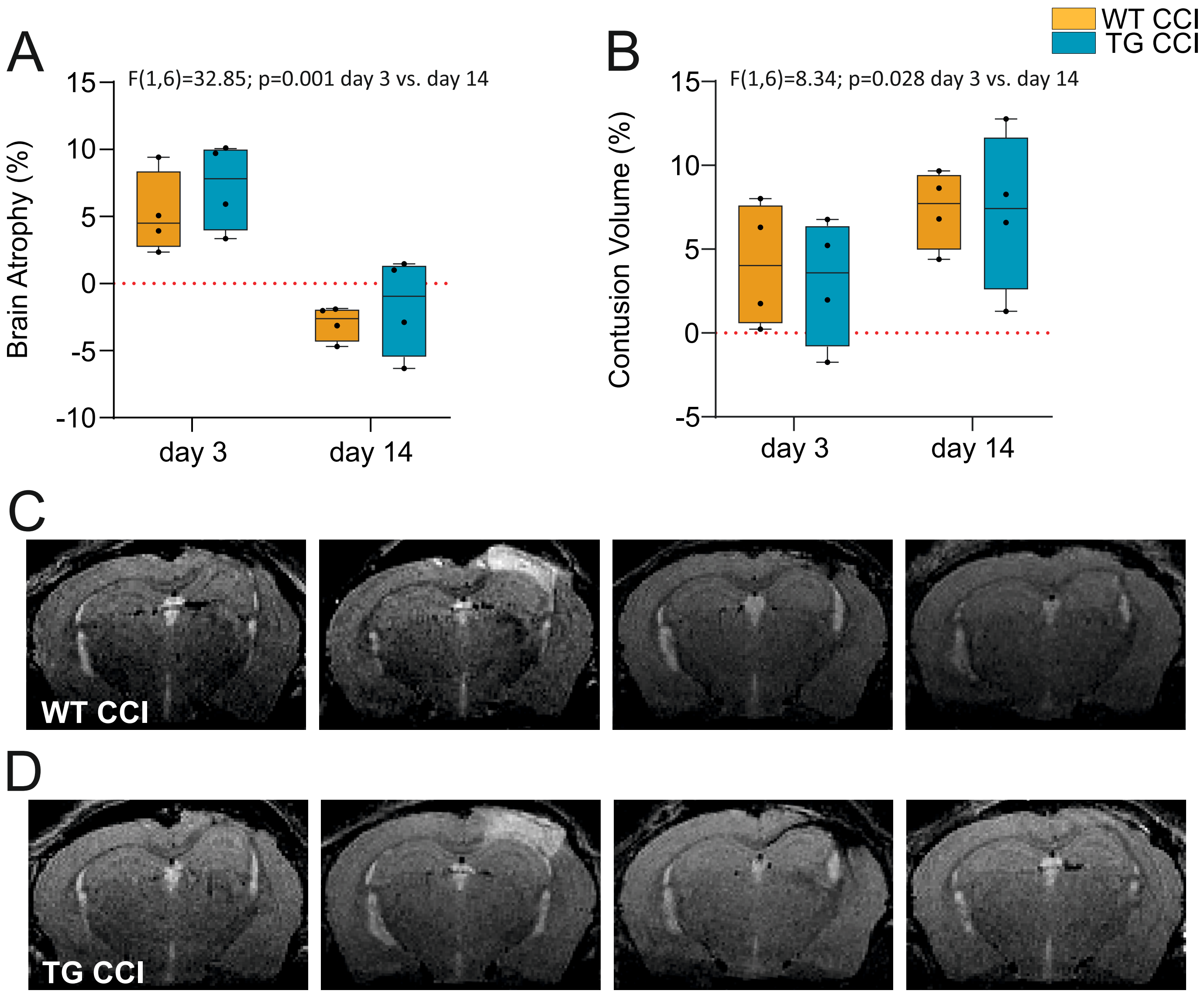

Supplementary Figure 5: Progression of anatomo-pathology in WT and TG mouse brains after induced moderate TBI, as observed by MRI **(A, B)**. Interestingly we observed a reduction in brain atrophy at 14 dpi, putative result of ongoing cytotoxic edema. No significant differences were observed between TG (n = 4) and WT (n = 4) mice. **(C, D)** Different types of lesions observed after CCI induction (21 dpi): WT CCI mice (C); TG CCI mice (D).
